# PALANTIR: An NFV-Based Security-as-a-Service Approach for Automating Threat Mitigation

**DOI:** 10.3390/s23031658

**Published:** 2023-02-02

**Authors:** Maxime Compastié, Antonio López Martínez, Carolina Fernández, Manuel Gil Pérez, Stylianos Tsarsitalidis, George Xylouris, Izidor Mlakar, Michail Alexandros Kourtis, Valentino Šafran

**Affiliations:** 1 Cybersecurity Department, i2CAT Foundation, 08034 Barcelona, Spain; 2 Department of Information and Communication Engineering, University of Murcia, 30100 Murcia, Spain; 3 Department of Information and Communication Technologies, Universitat Pompeu Fabra, 08018 Barcelona, Spain; 4 UBITECH Ubiquitous Solutions, 15231 Athens, Greece; 5 ORION Innovations PC, 11744 Athens, Greece; 6 Faculty of Electrical Engineering and Computer Science, University of Maribor, 2000 Maribor, Slovenia; 7 Sfera IT d.o.o., 2000 Maribor, Slovenia

**Keywords:** Security-as-a-Service, security orchestration, policy-driven management, virtual network functions, finite state machines, constraints programming

## Abstract

Small and medium enterprises are significantly hampered by cyber-threats as they have inherently limited skills and financial capacities to anticipate, prevent, and handle security incidents. The EU-funded PALANTIR project aims at facilitating the outsourcing of the security supervision to external providers to relieve SMEs/MEs from this burden. However, good practices for the operation of SME/ME assets involve avoiding their exposure to external parties, which requires a tightly defined and timely enforced security policy when resources span across the cloud continuum and need interactions. This paper proposes an innovative architecture extending Network Function Virtualisation to externalise and automate threat mitigation and remediation in cloud, edge, and on-premises environments. Our contributions include an ontology for the decision-making process, a Fault-and-Breach-Management-based remediation policy model, a framework conducting remediation actions, and a set of deployment models adapted to the constraints of cloud, edge, and on-premises environment(s). Finally, we also detail an implementation prototype of the framework serving as evaluation material.

## 1. Introduction

In recent years, threats such as ransomware have risen considerably (e.g., an 82% increase between 2020 and 2021). Large enterprises, as well as Small- and Medium-sized Enterprises (SMEs), have been attacked. Some of the prevalent threats in 2021 were cloud vulnerability exploitation, cloud service provider abuse, and credential theft [1]. SMEs are mostly affected by phishing, malware, credential theft, and ransomware, as identified in a 2021 survey [2], where more than half of the participating organisations were US-based, had less than 25 employees, and focused on manufacturing, healthcare, and retail. During that year, enterprises provisioned an average of USD 11.4m for IT security and spent USD 927k on remediating data breaches, whereas SMEs provisioned an average of USD 267k [3] and spent USD 105k to react to data breaches. This clearly illustrates that smaller organisations are largely exposed and have less leeway than larger ones, given that such attacks cost large enterprises the equivalent of roughly 8% of their total security budget, in contrast to just over 39% for SMEs.

In addition to the restricted budget availability, this trend is even more worrying for SMEs when considering that more than half of data breaches impacted SMEs in 2021 [4] and, furthermore, that the prevalent size of such organisations can be reduced to just a few employees (less than the aforementioned average of 25 in the US), depending on the country’s economic dynamics. In this sense, small and microsized enterprises have the losing hand due to their scarce resources and funds, which directly translates to a lack of availability of security expertise, and sometimes even in networking and IT itself.

Without adequate knowledge, such businesses can barely implement best practices to reduce the chance of attacks, are not likely to monitor their infrastructure for anomalous behaviour, and will not have the means to identify active threats and decide the best mitigation actions. In the end, smaller businesses can only rely on external outsourcing of security to secure their software and to make up for the missing knowledge and techniques while keeping cost at bay.

A possible option to reduce costs related to security outsourcing is to virtualise and automate the typical security life cycle, consisting of (i) continued monitoring to detect anomalies; (ii) the extraction of threat intelligence to determine the attack vector and behaviour; and (iii) the selection of the best measure to apply (security enabler and configuration) during the incident response. Cloud-related technologies, architectures, and standards are key enablers to achieve the aforementioned results, provided they are supported by adequate deployment models to access resources needing protection. For instance, the Network Functions Virtualisation (NFV) architecture is a good candidate to provide the substrate for the virtualisation of the security services and its automated deployment.

The NFV technology encompasses different standards that lay out the principles to adequately manage the network and provision the network services on top of it. Different families of standards compose NFV, such as Interfaces and Architecture (NFV IFA), Management and Orchestration (NFV MANO), or Security (NFV SEC). This architecture usually goes hand-in-hand in the literature with the Software-Defined Networking (SDN) paradigm, as both are complementary to realise the latest advances in networks such as 5G [5], Beyond 5G (BG5), and, predictably, also 6G.

The EU-funded PALANTIR project [6] is an innovation action from the European commission that targets the facilitation of the management of cyber risks and incidents by small and medium enterprises. The project provides threat detection capabilities, then shares such information with the countermeasure process, which capitalises on NFV by managing and operating security mechanisms that extend Virtual Network Function (VNF) instances [7]. These mechanisms are proposed in an as-a-Service market, allowing developers and security service providers to be remunerated for their activity.

### 1.1. Motivations

The cybersecurity life cycle entails the application of a diverse set of expertise and tools/techniques to (i) identify risks and assets at risk; (ii) to proactively monitor and detect anomalies; and to (iii) respond and mitigate the attack and threats; all to ensure business continuity. This process typically follows a conventional OODA loop (observe, orient, decide, and act) or a refined seven-step model, known as the Cyber Kill Chain [8], to bolster an organisation’s defences [9]. Within the cybersecurity life cycle, detection has been one of the most studied processes to date. However, monitoring has also been reinforced in recent years with the introduction of cutting-edge advanced Artificial Intelligence (AI) techniques that demand large amounts of data. In contrast, efficient mitigation of incident responses has been undervalued; studies have focused mainly on the implementation of well-known actions such as blocking malicious links, sandboxing potential attackers, or hijacking connections for emulation, to name a few.

Additional dimensions must be envisaged in order to secure the continuity of the business, such as those related to the coordination and deployment of countermeasures: for instance, (i) the deployment of the best enabler at the business’ disposal, both as reaction and also monitoring mechanisms for more effective detection; (ii) the identification of variables such as the risk associated with the organisation’s assets; (iii) the monitoring and analysis of capital expenditures (CAPEX) and operational expenses (OPEX) for financial sustainability, where an easily configurable system with minimal expert overhead will help to minimise OPEX costs; and (iv) the leverage of security services to offer a solution that fits many problems, unlike traditional managed Security Information and Event Management (SIEM) and Security Operations Centre (SOC) solutions. All of this must be achieved without overlooking the implementation of different monitoring and reaction enablers, following several deployment models adapted to the variety of resources to protect.

### 1.2. Contributions

In this article, we propose a security policy-based incident remediation methodology adapted to cloud, edge, and on-premises environments, which responds to an extensible range of cyber threats. Specifically, our contributions span across (i) delivering an ontology to specify and describe the properties and relationships used to select the operated security enablers, and how to financially follow their exploitation; (ii) a Finite State Machine-based policy for countermeasure procedures, ensuring the local scalability and consistency of the security enabler’s operation; (iii) a framework exploiting these contributions to conduct countermeasure actions; (iv) a set of deployment models for this framework to ensure its pervasiveness with the resources needing protection; and (v) the implementation and quantitative evaluation of such a framework.

### 1.3. Benefits

The adoption of the Security-as-a-Service (SecaaS) paradigm in PALANTIR makes it possible to propose consistent and idempotent remediation procedures, open to extend their threat coverage by deploying additional security enablers. Their operation is measured to ensure the financial sustainability for the subscribers and the security provider. Furthermore, the enactment of the security enablers is also tailored to the particularities of the environment’s required protection due to the availability of several deployment models, which permit technically adapted and pervasive protection. Finally, our approach also considers the privacy aspect of the deployment of security enablers, by documenting their impact on data confidentiality and integrity of the protected assets.

### 1.4. Organisation

The remainder of this paper is structured as follows. Section 2 analyses the related work. Section 3 presents two use cases to motivate SecaaS adoption and the use of PALANTIR. Section 4 exposes the overall architecture, detailing the main PALANTIR blocks and their characteristics. Section 5 explains the data modelling performed, giving special attention to the remediation process. Section 6 documents the testbed environment as well as the implementation and evaluation obtained. Finally, Section 7 lists the conclusions and suggestions for future work to perform in PALANTIR.

## 2. Related Work

This section describes first the general NFV idea and compiles some of the research efforts that leverage it for automated deployment platforms, as well as those explicitly applying it to address security concerns. Then, it covers those using policies to manage the security in virtualised environments. Finally, it summarises some findings from the literature and standards on the enforcement of the security actions.

### 2.1. NFV Usage for Cybersecurity

The European Telecommunications Standards Institute (ETSI) proposed, in NFV-001 [10], the NFV architecture, aimed at transforming the way networks are architected. NFV evolves standard IT virtualisation technology, consolidating or homogenising the management of the logic distributed across the infrastructure, i.e., a varying amount of network devices or high-volume servers; these can be located at different Points of Presence (PoPs), such as DCs, network nodes, or end-user premises. To that end, nine use cases were provided, targeting scenarios involving infrastructure, platforms, and software as a service, with others related to the virtualisation of mobile base stations or fixed access networks, among others. The final architecture was proposed in NFV-002 [11], and can be roughly divided into three main building blocks: (i) the deployed services as VNFs, (ii) the NFV Infrastructure (NFVI), and (iii) the NFV Management and Orchestration (MANO). Each Network Service (NS) contains one or more VNF, which virtualises logic in the form of VMs or containers and allows its internal management via an Element Management System (EMS). The second block (NFVI) is the physical substrate providing the logical resources, managed by the Virtualised Infrastructure Manager (VIM), to virtualise the NS instances with specific virtualisation; it also hosts other leveraged tools. Finally, the MANO is in charge of managing the life cycles of the NSs (mostly deployment- and configuration-wise) by appropriately instructing the VIM, and consequently overseeing the involved resources.

The application of the NFV framework is a challenge largely explored in the literature. For instance, the z-TORCH framework [12] presents automated NFV-based orchestration and monitoring reconfiguration mechanisms, which base its placement decisions on the profiling (carried out by Machine Learning) of Key Performance Indicators for the monitored NS instances. In the same manner, T-NOVA [13] proposes a software-based MANO stack for NFV-based infrastructures. This framework is focused on the NFVI resources, and develops a mechanism to assign resources, taking into account the particular characteristics and needs of the VNF workload. To that end, their monitoring system analyses the NFVI resources and the status of the NS instances. In edge scenarios, the LightMANO architecture [14] enables the deployment of a MANO framework in a massively distributed environment, exploring conditions such as the lack of resources available in the multiple scenarios where the system can be deployed. The LightMANO components are based on lightweight virtualisation technologies, such as containers; thus, the architecture is more suitable for the Multi-Access Edge Computing (MEC) paradigm. Another example is that of LASH-5G [15], which integrates service chaining for an end-to-end, VNF-based orchestration system to dynamically steer and adapt trafficdepending on latency and reliability needs.

When aiming to achieve high performance in network processing, the P4NFV architecture [16] allows deploying HW-accelerated Network Function (NF) implementations. This work argues that NF-based implementations are purely based on SW and, as such, they cannot achieve requirements for a large enough bandwidth (e.g., meeting a line rate processing target, mostly for small packets) or the optimal computing needs, if compared with specific-purpose implementations. P4 allows developers to create HW- and SW-based networking devices that P4NFV combines with the NFV architecture in order to use hybrid infrastructure resources. NFV is widely leveraged by 5G-based environments. For instance, Sanchez-Aguero et al. [17] explore the integration of two different and complex domains: (i) a 5G radio access network and (ii) Small Unmanned Aerial Vehicles (SUAVs). In this instance, NFV environments of different types are onboard the SUAVs, which are then deployed to serve 5G connectivity in remote or rural areas. Several other works combine Machine Learning (ML) techniques to augment the capabilities of NFV, whereas the NetML framework [18] instead uses NFV as a means to optimise the behaviour of ML applications. NetML benefits from the usage of libraries and tools to accelerate packet processing workloads, and from the adequate management of CPUs and GPUs in Common Off-The-Shelve (COTS) hardware, achieving reduced latency and increased data transmission to ML applications through a Direct Memory Access engine incorporated into GPUs.

Finally, NFV has also been explored previously as a lever for infrastructure protection. For instance, the H2020 SHIELD project [19] proposed a cybersecurity framework based on virtual Network Security Function (vNSF) instances, deployable as services to protect the network layer of the subscribers’ companies. Pattaranantakul et al. proposed SecMANO [20] to extend the ETSI NFV MANO in order to orchestrate and enforce high-level security policy on the network infrastructure. From an industrial perspective, ETSI published the NFV-SEC 013 group specification [21] to cover the design of a security management plan for NFV environments.

It can be observed that NFV (along with other architectures, frameworks, and languages) led to another step in the cloudification of the network. NFV also aids in the convergence of networking and computing functions by abstracting their management in the Everything-as-a-Service (XaaS) approach in virtualised substrates. The evolution of such convergence across network domains and resource management can be seen from pioneer projects such as T-NOVA or UNIFY to initiatives such as CORD, as expressed in [22]. In this sense, the approach proposed in this paper leverages some benefits provided by previous works, extending the NFV framework to cover the protection of the endpoints by following a SecaaS approach spanning both networking and computing levels, whilst considering the protection of both the edge and the private and public clouds.

### 2.2. Policy-Driven Security Management

Policy-based management has enabled the automation of resource management in cloud and edge environments. For instance, Tsagkaropoulos et al. [23] explore how the OASIS Topology and Orchestration Specification for Cloud Applications (TOSCA) language can be extended to cover the deployment of distributed applications in the cloud–edge continuum. To address the security usage, this language can be extended to incorporate security aspects, such SecTOSCA introduced in [24]. However, similar to SecureUML [25], SecTOSCA emphasises the secure design of the resource needing protection and does not cover remediation procedures, in contrast to our work. Capturing non-complicated high-level requirements to generate extensive policies is a challenge tackled by intent driven policy management. This approach is actively prospected by the software network community, and has been successfully transposed to security issues, as INTPOL [26] proposes. However, it remains mainly centred on programmable switches, while our approach targets a wider spectrum of resources to operate.

A major challenge in elaborating a policy model for security is ensuring its fit-for-purposeness regarding the nature of the resource to protect and the security feature to deliver. For instance, the eXtensible Access Control Markup Language (XACML) [27] is a reference policy model and architecture for access control. In [28], the author proposed a high-level policy language and architecture to supervise DDoS attack mitigation. The authors of [29] introduced the X2CCDF language to first evaluate the configuration exposure of a set of operated resources according to a security checklist, and then to determine a sequence of remediation actions exploiting first-order logic. However, the scope of these works is limited to a single specific security feature. Regarding the environments to protect, T-Cloud is an access control framework [30] aimed at the protection of resources deployed in cloud environments. The particularities of the Internet of Things (IoT) environments has also fostered the emergence of specific ontologies, such as those described by the authors of [31]. Preuveneers et al. [32] also operated software assets in their work through container-based services. In our case, in PALANTIR, we endeavor to model remediation policies framing different security features and landscapes of assets to protect by proposing an extensible policy format and delegating the technicality to the mechanisms.

### 2.3. Security Enforcement

Enforcing security constraints on a resource, in order to protect or remediate a threat, induces several challenges. For instance, the resources to be protected may require some particular form of management, which may be complex to coordinate and which may not be met by a constrained resource. Previous work [33] has been conducted specifically to introduce the IoT into the scope of SOCs. The software-defined security concept [34] sets out to uncouple the security management from the resources needing protection to propose unified security supervision. In [35], the authors applied this approach to multi-tenant and multi-cloud infrastructures by leveraging the programmability of security enforcers.

In PALANTIR, we capitalise on the aforementioned approach and extend it from cloud to mobile edge computing and on-premises environments. A prerequisite to leverage this strategy is to employ a normalised interface between the management components and the security mechanisms enforcing security properties. The software network community has considered extending the management interfaces to meet security management objectives. For instance, ETSI published the specification NFV-SEC013 [21] for security management and monitoring. In RFC 8329 [36], IETF considers programmable network security functions to enforce security policies as part of the Interface to Network Security Functions (I2NSF) and defines a reference model for it. In PALANTIR, we consider exploiting such interfaces to meet security objectives not limited to network infrastructure, but that also cover typical information system assets and devices.

Finally, the pervasiveness of security decisions is also an issue, especially when considering the increasing complexity of information systems. The adoption of the software-defined paradigm in areas beyond networking [37] has enabled a new perspective to tightly enforce security constraints via programmability. At the infrastructure level, Hedi [38] evaluated the security of hypervisors and their intercommunication, while Snappy [39] programmed in the Linux kernel to enforce security policies for containers based on extended Berkeley Packet Filter (eBPF). In PALANTIR, the pervasiveness of the security environment is tackled by coupling a set of specialised mechanisms with deployment models, representing the environment where resources to protect are instantiated.

## 3. Motivating Cases

Two of the use cases developed in PALANTIR are presented below to motivate and demonstrate the protection capabilities developed for their implementation as SecaaS solutions. The first use case is geared towards the protection of assets in a healthcare environment, while the latter focuses on an e-commerce platform. This section concludes by outlining a number of requirements to consider when exercising the remediation procedure.

### 3.1. Use Case 1: Protection of the E-Health Environment

The digitisation of healthcare brings with it an inevitable surge in cyber attacks. As a consequence, the healthcare industry is especially at risk due to (i) the value of sensitive Personally Identifiable Information (PII) housed within systems; (ii) an increase in the size of the Internet of Medical Things (IoMT); (iii) insufficient cybersecurity protection; (iv) the need for data transparency; (v) and ineffective employee awareness training. In return, this produces an increasing concern over cybersecurity threats that challenge their operations. One of the primary concerns are ransomware attacks that may disrupt operations, decreasing the ability to maintain the quality of patient care; this is in addition to the financial implications of the ransom, such as the cost of remediation, or the reputational damage. According to the survey conducted by H-ISAC in 2021 among executives in healthcare, the top three threats in 2021 and 2022 were ransomware, phishing, and data breaches [40].

In the above context, the deployment of SIEM solutions is expected. SIEMs improve incident detection, speed up incident management, and enforce adherence to specific regulatory compliance needs [41].

### 3.2. Use Case 2: Resilience of the E-Commerce Platform

Although the new generation of SIEMs delivers powerful features in terms of storage, visualisation, and performance, as well as the ability to automate the reaction processes, the available countermeasures are limited, static, and deployed without comprehensive consideration of the impact of countermeasures on the infrastructure or even the continuity of the business [42]. Furthermore, cyber threats targeting SMEs vary significantly from threats in the large-scale industrial sector (for which most SIEM solutions have been designed [43]). For instance, phishing and malware present critical issues, especially for the SME sector. Furthermore, traditional SIEMs require the use of additional, external solutions to automate the remediation. In fact, although over 51% of SMEs use AI for detection, only 18% of companies use AI/ML solutions in remediation [44].

Finally, since ML methods have to adapt exactly to the respective environment, modern IT systems require a vast initial investment of personnel time as well as a high degree of expertise. However, in SMEs and Microenterprises (ME), IT support is often seen as cost-centric rather than a revenue generator [45]. Thus, SMEs/MEs focus on the continuity of IT services rather than protecting the key information resources and infrastructure. Furthermore, over 55% of SMEs and MEs lack the expert knowledge required to understand the risks and implications of policies such as "bring your own device" or the lack of an up-to-date cyber risk strategy poses to their business [46]. With this use case, we integrate the PALANTIR platform into the cloud and on-premises ecosystems of an ME involved in e-commerce and, in particular, an ecosystem with physical offices and a cloud environment. Each physical office consists of several residential-grade IT pieces of equipment (e.g., routers, low-cost switches) and various (personal) devices connected to the internet. Access to virtual space (e.g., PLESK platform) and (web) services (e.g., SQL and no-SQL databases, FTP server, web server) is protected with an Account Restriction policy via User Roles. Within the virtual space, the ME implements and runs its Customer Relationship Management (CRM), e-retail store, mail servers, product and content personalisation, and a Small Business Support System (BSS) framework to improve efficiency and flexibility. The majority of personal customer data are stored and handled in the cloud. The timely backups, malware protection, Bot (e.g., SEO spam and injection attacks), DoS, DDoS, and brute force attacks represent the major threats to e-commerce solutions [47].

### 3.3. Requirements of Remediation Procedures

When considering the threats indicated above, the involved components impose several requirements for the remediation procedure, acting as guiding principles at different levels. These requirements are also faceted by the SecaaS paradigm, contributing to the extensibility and openness of the security mechanisms enacting these procedures. In order to properly deliver the remediation procedures, the Security Capabilities (SCs) are implemented as one or more tightly combined security mechanism(s) following the SecaaS paradigm. This follows a design that abstracts the SC Hosting Infrastructure (SCHI) in which the SCs will be deployed and configured, being generic enough not to depend on specific deployment configurations other than those directed by the platform itself. The *idempotence* is another requirement that the SCs and the Security Orchestrator (SO) must honour, since any re-enactment of the exact same previously deployed remediation procedure shall not further modify the environment after the first attempt.

The abstraction tenet governs the orchestration layer at different levels. For instance, while the MANO can generalise part of the underlying layers by leveraging multiple integrations with the virtualisation management tools and those abstracting the cloud-based services’ configuration, it also requires some low-level details on the infrastructures where the SCs will be deployed. In this regard, the SO also requires access to the overseen infrastructures to allow the extraction of information and monitoring of the clusters, as well as to enable the enactment of some remediation procedures directly in the virtualisation nodes.

On the substrate layer, at the infrastructure level, the SCHI is naturally required to provide compatible virtualisation resources for the MANO to deploy the SCs. It must also support proper connectivity between the MANO and each virtualisation management process, since this will allow the security functions to reach the affected system(s).

## 4. PALANTIR General Architecture for Threat Remediation

The use cases defined previously heavily influence and guide the behaviour of the PALANTIR platform at two levels: (i) the architecture implemented by the platform; and (ii) the deployment models, which tailor the platform deployment to the customer needs and/or restrictions. First, a general overview of the architecture is provided, comparing it to that used for pure network operation, specifically for 5G environments. Then, details are given for the remediation actions and their notification to end-users, as well as the selection, availability, orchestration, and design and operation themselves for the SCs. Finally, the deployment models are described and compared against each other using a set of dimensions or characteristics deemed specially relevant for this case.

### 4.1. Overview of the Architecture

**Figure 1:**
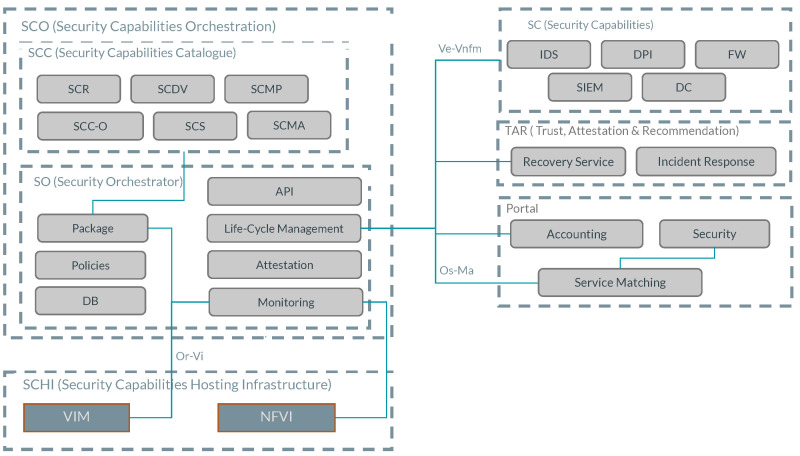
PALANTIR general architecture.

Figure 1 outlines the PALANTIR general architecture. SCs are the protection mechanisms (security enablers) of PALANTIR, being managed and deployed by the SO into the SCHI to realise their function.

On the other hand, the SCHI is composed of the VIM and the NFVI. The SCs are interfaced by the SO via the interface *VeNf-Vnfm*, the component in charge of the SCs (i.e., the SecaaS solutions) life cycle management. The SO incorporates some key elements such as Monitoring and Package, explained in detail below in Section 4.4. The SO is connected to the SCHI to correctly instantiate SCs and the SC Catalogue (SCC), understood as the SC repository with the descriptors and metadata to deploy whichever SC is registered in PALANTIR. The SO and the SCC maintain a tight bond to shape the SC Orchestration (SCO).

Accordingly, the PALANTIR platform has the Trust, Attestation, and Recommendation (TAR) component, where the Recovery Service (RS) and the Incident Response (IR) are hosted. These two components monitor the SCHI and SCs and report alerts/messages with remediation procedures to mitigate threats. Finally, The PALANTIR Portal is the connection with the final user, that is, the information point used by the client in order to know the current status, in real time, of the infrastructure. The Portal is also in charge of the Accounting section, and includes Service Matching (SM) as the component with the proper knowledge of selecting given SCs under certain conditions and the results obtained. The PALANTIR Portal is connected to the SO via the *Os-Ma* interface to receive notifications about the SCs life cycle and other events occurring in the SCHI.

**Figure 2:**
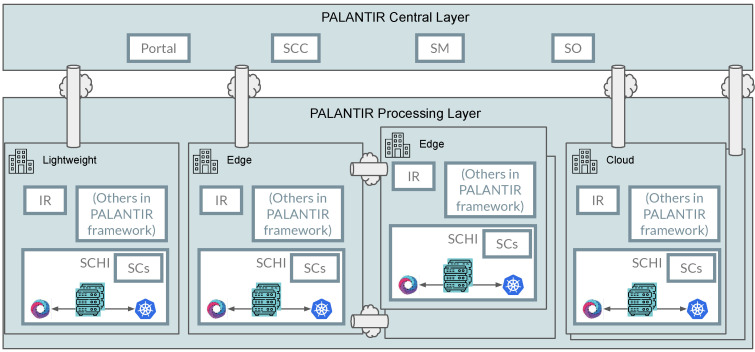
Deployment models foreseen by PALANTIR.

When compared to the 5G’s Service-Based Architecture (SBA) [48], the PALANTIR architecture also (i) separates the user plane (client’s infrastructure) from the control plane (central layer), where the latter hosts common core functionality, as presented in Figure 2; (ii) provides a central repository (the SCC) that allows the system to expose new network functions in the same fashion as the Network Repository Function (NRF) in SBA; (iii) enables SC instances (as NFs) to be exposed and consumed in a loose manner and without impact on other existing SCs of NFs; and (iv) enables inter-NF communication (or from other applications), as the functionalities implemented by the SC instances are exposed via the SO and accessed through TLS channels (e.g., to request data from another SC instance or configure it, as required by the encompassing network service). All of this partially enables the logic devised for the Network Exposure Function (NEF) in SBA. On the other hand, and different to SBA, (i) the SC instances (as NFs) are not considered as part of the control plane, but are instead deployed and operate in the user plane, covering the client’s infrastructure; (ii) the core functionality is not restricted to acting in the network, and thus is not designed as NFs, but nevertheless follows a Service-Oriented Architecture (SOA), suited for heavy integration.

### 4.2. Coordination of the Remediation Procedure

When a threat is detected and classified by an external detection tool, the IR component is notified with the main characteristics of the threat to handle. The devised reactions may comprise either the reconfiguration of existing SCs in the managed networks or the deployment of new ones, as well as the exploitation of other services in the PALANTIR infrastructure. The IR component is able to adapt the generic operations defined in the recipes to the status of the network, such as the presence of a specific SC needed to deploy a recipe (e.g., the iptables packet filter). The PALANTIR-protected infrastructure hosts multiple IR instances that are responsible for triggering mitigation policies, right after a threat/attack related to data breach is detected. The objective of the IR is to handle remediation policies for threat mitigation, especially those that cannot be handled by, e.g., the cross-system or stakeholder notifications, which may be specific to an individual entity (SME/ME). IR implements predefined Finite State Machines (FSMs) to tackle specific incidents. This approach, and those handling personal policies, are explained in Section 5.2.

When a threat is detected and classified by an external detection tool, the IR component is notified with the main characteristics of the threat to handle. Reactions may comprise the reconfiguration of existing SCs in the managed networks, the deployment of new SCs, or the exploitation of other services in the PALANTIR infrastructure. The tool is able to adapt the generic operations defined in the recipes to the status of the network, such as the presence of a specific SC needed to deploy a recipe, e.g., the iptables packet filter. PALANTIR-protected infrastructure hosts multiple IR instances that are responsible for triggering mitigation policies when a threat/attack related to data breach is detected. The objective of the IR is to handle remediation policies for threat mitigation, especially those that cannot be handled by, for instance, the cross-system or stakeholder notifications, which may be specific for an individual entity (SME/ME). IR implements the predefined FSMs to tackle with the specific incident. The usage of the FSM approach and how IR handles personal policies is explained in Section 5.2.

### 4.3. Selection of the Security Capabilities

When conducting remediation procedures, deploying an additional security mechanism may be required to obtain the needed mitigation capacities, or extend the detection feature for better threat qualification. Nonetheless, selecting the adequate mechanism requires to account for the technical constraints conveyed by the SCHI. Namely, the mechanism to be deployed should be (i) compatible with the adopted deployment model; and (ii) do not exceed in resource requirements what the SCHI can sustain. From a financial standpoint, the suggested deployment should also minimise the cost induced by the mechanisms operation.

The SM is in charge of this selection process. Specifically, from a list of security requirements to be enforced issued by the IR, the SM will determine a list of implementations to be enacted, and schedule them on the adequate architecture. The selection process has been modelled as a constraints-satisfaction problem that we detail in Section 5.3.

The SM will first interface with the IR through message-oriented middleware to receive the deployment request comprising the security requirements to enforce. Then, the component queries the list of available infrastructure resources to the SO to understand which SCHI resources are available for deployment. The SCC conveys the list of mechanisms available for deployment and their characteristics. According to the information gathered, the SM populates the model by exploiting the constraints engine solver, and initiates the solving process. Eventually, if the solver narrows down one or several solutions as deployment plans, the SM interfaces with the SO to trigger the deployment aligned with the cheapest solution. If no solution is found, a notification is broadcast to justify the issue to a human operator. This specific case happens when no SC is available to support the requirements or when no compatible infrastructure resource can support the SC to deploy.

### 4.4. Availability of the Security Capabilities

The SCs are registered within a central catalog, that is both, secure and searchable. The SC packages, along with accompanying metadata, are registered by certified SC developers and are safely stored in the SCC.

The SCC stores the metadata of the SC packages in a trusted way, by keeping the SC software image references, the security and privacy specifications, billing information, and other (VNF) operational metadata.The metadata, along with integrity indicators, are stored in the SCC database. The stored SC packages can be directly used to instantiate (onboard) the SCs by the SO. The packages include the software of the SC alongside the operational and attestation metadata. The software of the SC is packaged by following the specification dictated by NFV MANO for NSs and Virtual or Cloud-native Network Functions (xNFs).The xNF and NS packages are stored in the SCC block storage. The packages also include operational and security descriptors. All SCs in the SCC can be searched for or filtered based on the metadata. By making search queries based on stored metadata, the proper SCs for monitoring or remediation can be identified by searching for SCs and correlating with the outcome of the analysis the SM conducts

### 4.5. Orchestration of the Security Enablers

The orchestration of the life cycle of the SCs is necessary for the SCs to act as Security Enablers, enacting the security functions or controls on a specific section of the managed infrastructure. The SO is the architectural building block that takes over this, acting as a central point that oversees the functioning of the SCs; extracting and concentrating information on both the available SCs and infrastructures where to deploy these. This introduces a logical layer that provides meaningful information to the rest of architectural building blocks from the platform, from a single access point and in a simplified manner prior. The interacting components can then use it to analyse a given status, select a given course of action and finally apply some action; where the action can be taken by another component or requested back to the SO, ultimately forming a feedback loop that can apply self-protection actions. Similarly, this layer can incorporate heuristics and internally enactactions on the infrastructure itself, where required to maintain the security of the overseen infrastructure. Finally, monitoring extraction and event notification are also triggered from this point to reach other components.

As depicted in Figure 1, the SO interacts with the SCC so as to both upload (or onboard) new SC packages and extract later specific metadata contained in them, as well as with the SCHI for operations such as (i) requesting the deployment of a SC on a given infrastructure; (ii) enacting Day-0, Day-1 or Day-2 actions on the running SCs (understood as cloudbased actions that are triggered during instantiation, boot and run- time, respectively); (iii) deleting the SC instance; or by (iv) directly acting with the SCHI servers to either monitor their status or, potentially, directly execute security-related actions on them (e.g., isolate a given cluster).

To accommodate the functionality explained above, the SO builds on top of tooling in the substrate layer. This layer is the SCHI, which comprises all resources that the infrastructure offers for the management of the SC running instances (typically, resources such as RAM or CPUs, which can be virtualised and accessed via containers or other virtual elements), as well as to physical resources that can be accessed to complement the functionality (e.g., crypto chips to assess integrity, such as the Trusted Platform Module (TPM) [49]) or tools related to infrastructure management (e.g., hypervisors such as KVM) or its orchestration (e.g., a Kubernetes cluster). The SCHI hosts virtualisation tools such as OpenStack, where each of them is considered VIM in the ETSI NFV terminology; alternatively, the NFVI hosts any other tools to support the operation. In addition to the SCHI, the SO is tightly integrated with an implementation of the NFV MANO component in the ETSI NFV architecture, named Open Source MANO (OSM) [50]. The MANO brings the orchestration logic to manage and oversee all the deployed NFV-based services, providing the SO with built-in integration with different VIMs such as OpenStack, as well as with tools in the NFVI that relate to (i) orchestration, typically Kubernetes (K8s) either running at public or on-premises clouds; and (ii) cloud frameworks that can, amongst others, enact the Day-0, Day-1 and Day-2 cloud-based configuration actions (i.e., via Juju [51] charms). In this integration with the ETSI NFV MANO, some ETSI NFV interfaces (or reference points) are specially relevant, as shown in Figure 1): (i) the *Or-Vi* allows the MANO to communicate with the resources in the SCHI, whereas (ii) the *Ve-Vnfm* enables it to remotely execute the cloud-based configuration actions on the running SC instances. The latter interface can also be leveraged to provide inter-SC instance communication in a loose and easy manner. Roughly speaking, it could be somewhat compared to the *Nnef* Service-Based Interface (SBI) from the control plane in SBA.

### 4.6. Design and Operation of Security Enablers

The SC architecture features two main modules: the Security Element Manager (SEM) and the xNF. The xNF is the recipient that contains the logic of the security enabler (e.g., a firewall), whilst the SEM connects the internal xNF and the PALANTIR components. Practical benefits of the SEM are the management of the life cycle and the configuration of the SCs, but also the exposure of the internal data collected in the xNF (e.g., for SCs detecting threats or forwarding relevant data from the client network to other PALANTIR components). When deployed, SEM and xNF are allocated together into an NS, which is the minimal deployment unit that the SO (and, in general, the underlying MANO) can understand. Finally, the NS is instantiated in a container-based SCHI, such as one deploying containers on top of K8s.

Essentially, PALANTIR covers two types of SCs to implement detection and mitigation capabilities: (i) mechanisms collecting data and monitoring network/host traffic to detect threats and anomalous behaviours; and (ii) security enablers with capacities of mitigating threats. The first type typically encompasses Intrusion Detection Systems (IDS), Deep Packet Inspection (DPI), and Data Collectors (DCs), whilst the latter may contain the Firewall and Router (FW) and SIEM solutions (e.g., Wazuh).

Each SC exposes interfaces against the PALANTIR platform, where the most relevant is that between the SO and the message (event) broker shared between the components in the PALANTIR platform. On the one hand, the connection with SO is used to manage the SC life cycle and trigger Day-0, Day-1 and Day-2 cloud-based configuration actions, as detailed above. To that end, each SC implements an operator container (through the Juju tool) that establishes a connection towards the xNF to execute commands and apply configurations to it. On the other hand, the SC sends relevant information for the PALANTIR components to a shared message broker. Such information is provided by the monitoring SCs (generated alerts) and via collectors’ SCs (retrieving sniffed traffic by means of NetFlow data, Zeek data, etc.).

### 4.7. Remediation Awareness

Security operators and end-users who are members of a client organisation need to be informed of the security-related events occurring in the network. As such, the PALANTIR Portal has real-time monitoring as a core functionality, and the ability to check past threats.

All the authenticated users who operate as members of client or PALANTIR provider organisations receive live notifications from the platform as they occur. The alerts and notifications are pop-ups at the top of the screen, regardless of the view they are currently in. The alerts and notifications originate from the PALANTIR platform as a whole, as well as the SCs their organisation has subscribed to. These pop-up notifications are colour-coded based on severity, with low-severity or simple notifications being green, medium-severity alerts being yellow, and critical or operation failure notifications being red. Unknown severity incidents or generic notifications just have a grey background. If the notification pertains to an incident, it is kept in an incidents page, and the pop-up contains a link to it in that view/page. In general, any notification that pertains to an element that is viewable somewhere on the dashboard in more detail, or is managed or controlled through a view system, then the notification contains appropriate links. Mitigation and remediation actions appear as notifications, stating briefly the kind of action that was performed on the network, along with the threat in response to which this action has been undertaken. The link to inspect the remediation action leads to the details of the threat and the corresponding remediation.

The dashboard also provides a consolidated view of past threats, including the applied remediation for each. Each remediated threat is indicated in the list of past threats. Viewing the details of such an element will show the details of both the detected threat and the remediation steps taken.

Information that is intentionally absent from the UI is remediation action prompts. Indeed, the PALANTIR platform strives to minimise the required human interaction, while ensuring network and systems integrity. This is conducted in order to ensure a faster response than the one CIRTs or CSIRTs comprised of humans would provide. As such, focus is given to (a) optimising the incoming threat and remediation action notifications and (b) providing a compact but detailed view of past threats and applied remediation actions.

### 4.8. Deployment Models

To further tailor to different customers’ needs, PALANTIR foresees three Securityas-a-Service-based deployment models related to lightweight (on-premise), edge, and cloud scenarios.

These are depicted in Figure 2, showing two main layers:

- *Central layer:* First, and common to all deployment models, the upper, central layer introduces the PALANTIR components that have some degree of observation of the overall procedures, e.g., those requiring support for multi-tenancy (both regarding customers, as organisations, and their infrastructures). These are the Portal, the SCC, the SM, and the SO.
- *Processing layer:* In contrast, the lower, processing layer shows the components running at a site local to or near the deployment infrastructure, used to instantiate the SCs and to operate closer to the infrastructure and captured data. These include the IR, the SCHI, the deployed SCs, and other components of the PALANTIR framework that are not described in this work.

Firstly, the Lightweight deployment model defines the minimum PALANTIR architecture to deploy as a Customer Premises Equipment (CPE) in the client infrastructure itself. This CPE includes the infrastructure (SCHI) and running SCs, as well as other PALANTIR components that ensure the correct operation in a PALANTIR-in-a-box approach, such as the IR component. Within this deployment model, a minimum SC service chaining is defined to deliver a functional block with filtering, IDS, and SIEM capabilities. It is worth noting that the filtering SCs shall be allocated and will act as the client router to ensure proper operation.

The Edge deployment model delivers the PALANTIR capabilities following a Mobile Edge Computing (MEC) paradigm in the network edge. This approach allows PALANTIR to provide protection to multiple tenants, and is mainly designed for 5G scenarios. In this case, PALANTIR favours deployment at the network edge, leveraging the increment of resources available, in contrast with the Lightweight deployment model.

Finally, the Cloud deployment model covers scenarios that can combine different clouds used by the client. Here, the SCs can be deployed in one of the available cloud infrastructures.

**Figure 3:**
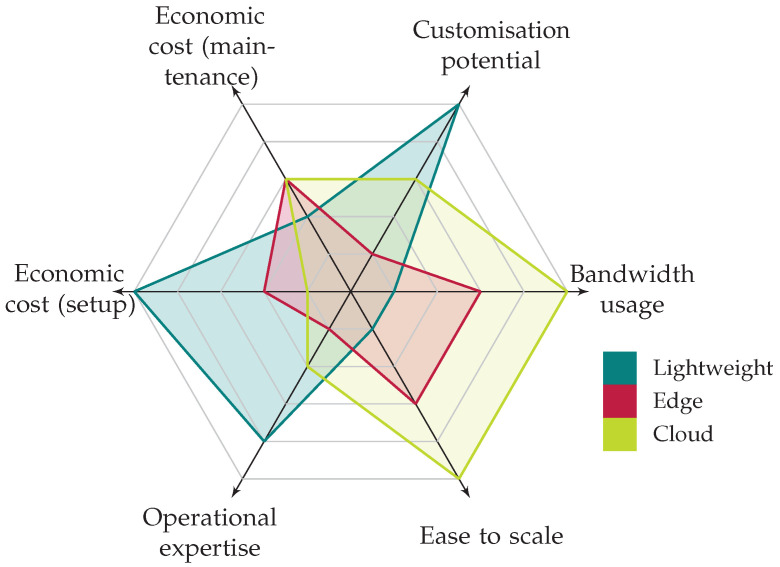
Comparison across deployment models.

A preliminary, qualitative comparison across each deployment model is given in Figure 3, which represents the perceived degree of fulfillment of a set of dimensions of interest (i.e., desired features) in every deployment model, and from the perspective of the end-user, whose organisation is being protected. Some of these may be expected to be minimised (bandwidth usage, all forms of cost, the required expertise needed to operate), whilst others should be maximised (customisation potential and the ease to scale). In this sense, Chi et al. [52] provided a comparison between public and private clouds, introducing characteristics that partially overlap with the dimensions here considered, as well as extending with others and providing the modelling and formulation for their analysis.

The Lightweight scenario fares best regarding its degree of customisation and the reduced bandwidth usage, products of most operations occurring onsite; however, this is at the expense of a high down payment to setup the required equipment and the need of a medium-to-high degree of expertise to setup the networking and possibly to understand some of the concepts of the configuration of the platform to be deployed. On the other hand, both the Edge and Cloud scenarios may fare best in terms of economic cost, regarding both the initial setup (which may be understood to be somewhat higher for the Edge scenario if assuming the higher scale at which the public cloud operators work compared to some network providers) and the maintenance cost. In both cases, operational expertise required by the user is relatively low. There is a perceived higher degree of customisation in the Cloud scenario because of the mature revenue channels and interfaces provisioned by the public cloud operators, in contrast with that of network providers. The bandwidth usage is assumed to be potentially more moderate in the Edge scenario than in the Cloud scenario, and considerably higher when compared to the Lightweight scenario. Finally, and similar to the perceived cost for the initial setup, the Cloud environments should provide easier means to scale compared to both the Edge and Lightweight environments.

## 5. Data Modelling

For proper understanding and application of measures, PALANTIR first modelled a general, platform-wide ontology that comprises all abstract entities being governed and establishes their foreseen interactions. Moreover, and specific to different components in the architecture, modelling their behaviour helps towards establishing more thorough, consistent behaviour. This is the case for (i) the remediation actions, each of them requiring a specific workflow; (ii) the SM to appropriately select the SCs that can best remediate a given identified threat; (iii) the considerations to be taken by the billing model, depending on the type of operation; and (iv) the internal definition of the data and metadata per SC (SC entry model), as maintained and managed by the SCC.

### 5.1. Ontology

Complete data modelling allows PALANTIR to act consistently and exhaustively against different threats and anomalies. In this context, the ontology defines the information used by the involved elements in different processes and tasks. Thus, the ontology is required to perform correct remediation procedures. The final intent of this ontology is to best understand which security mechanism to leverage under certain conditions, looking for the CAPEX and OPEX financial sustainability from a client’s perspective.

**Figure 4:**
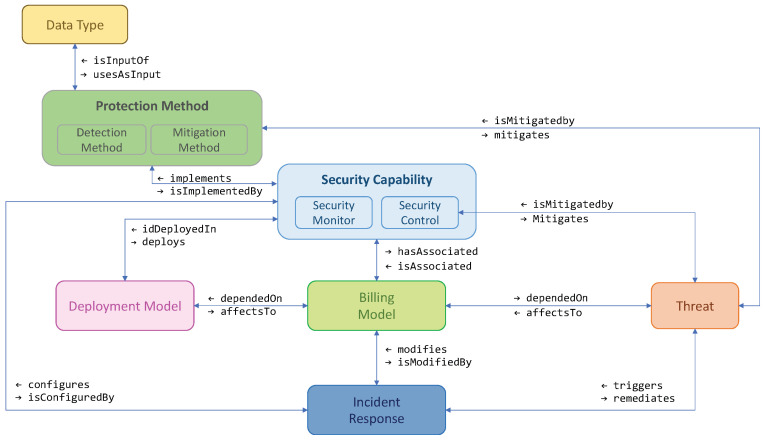
Ontology defined for the formalisation of data in the PALANTIR remediation procedure.

Figure 4 depicts the ontology with its different classes and relations. It contains seven classes, which relate to the main PALANTIR components involved in the remediation procedures. Their significance and relationships are described below.

- The *Data Type* represents the different data modalities contemplated in PALANTIR (e.g., NetFlow and Zeek data).
  This class is connected to the *Protection Method*, and uses the latter as input.
- The *Protection Method* class defines the detection and mitigation methods (i.e., the two types of available protection) as its subclasses.
  It is connected to two specific classes: *Security Capability* and *Threat*.
- The *Security Capability* class is the formal name for the security mechanisms, and it refers to the virtual element developed to be deployed in the client infrastructure.
  This class supposes a central part of the ontology because it has a relationship with a large portion of the ontology classes. Section 5.5 delves into details about the properties and metadata modelling this class. In addition, a *Security Capability* can implement one or more protection methods and is related to the *Threat* class, contributing to mitigate the effects represented by the latter.
- The *Threat* class defines the threats/attacks following the indications of threat modelling methodologies such as OCTAVE [53] and STRIDE [54], well-known tools for representing such types of activities.
  This class is also connected to the *Billing Model* and *Incident Response* classes because it directly affects the data allocated among both classes.
- One the one hand, the *Billing Model* class represents the different fees to be applied regarding the deployed SCs and the contracted characteristics.
  The connection between the *Billing Model* and *Threat* classes is due to a *Threat* generating a remediation procedure that deploys a new security mechanism. This fact is also represented by the connection between the *Billing Model*, *Incident Response*, and *Security Capability* classes.
- In addition, the *Billing Model* depends on the *Deployment Model*; the latter defines the deployment models available, which have associated different physical resources, operation modes, etc.
- Finally, the *Incident Response* class models the element selecting and performing the remediation procedures when a threat/attack is detected.
  Therefore, it connects with the *Threat* and *Billing Model* classes, already described, but also with the *Security Capability* class, since the remediation procedure to be performed can result in the reconfiguration of a security mechanism deployed in the client infrastructure.

The ontology presented in Figure 4 outlines the definitions of the different PALANTIR operations involved in the remediation procedures. This is the case of the SM, where the following flow can be defined: a flow *Threat* triggering the *Incident Response*, modifying the *Billing Model* (depended of *Deployment Model*), and configuring *Security Capability*, which mitigates the *Threat*.

### 5.2. Remediation Policy

**Figure 5:**
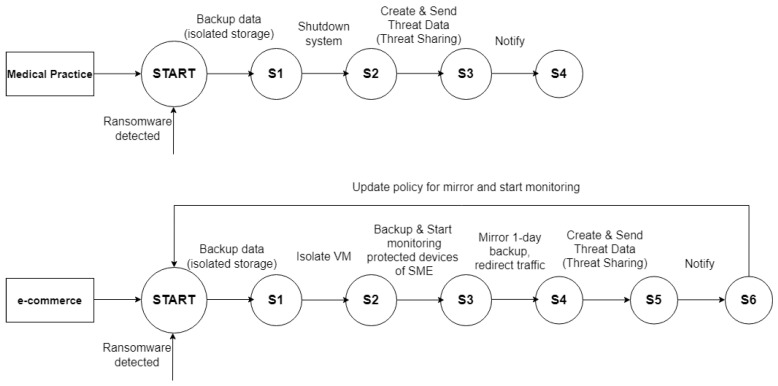
Examples of a personalised policy presented as FSMs.

Remediation policies can be designed intuitively as flow charts (Figure 5) using a graphical interface. In order to be executed by IR, these are transformed and stored as Unified Modelling Language (UML) files. Using the UML methodology, we ensure unified design, clean structure, and portability in order to easily include the created remediation policy in the IR instances. The FSM approach allows the creation of personalised and highly flexible remediation, which is deterministic. The outcomes are predictable and easily verifiable. Namely, the remediation steps are limited to a finite set of possible paths and states. Although being simple to comprehend and put into practice, an FSM is frequently disregarded.

Despite significant advances in security enforcement, attackers are still able to compromise organisations’ computing resources [55], explained by the increasing management complexity handled by security teams [56]. In our approach, the FSMs open the opportunity to formalise and personalise the policy decisions related to user behaviour, organisational systems, and data in all access contexts. A policy is automatically exported and integrated into the IR, where it waits for activation by an outside trigger, i.e., the definition of a newly detected cyber threat. Figure 5 showcases two FSMs designed to trigger personalised remediation actions for use case 1 (e-health in the medical practice) and use case 2 (e-commerce). As outlined in Section 3, the context of the two use cases is slightly different. Namely, in the case of a small medical practice using an e-health solution, we assume a fully local infrastructure with one server being used for the practice (i.e., storage and operation). In e-commerce, however, the operational context is distributed across a cloud environment (delivering services, including storage) and local devices (PCs using such services). Furthermore, in medical practice, the end-users are doctors and nurses or administrators, whereas in e-commerce, the end-users are employees and citizens. Finally, the priority related to data protection and the operational environment is also different. Namely, for e-commerce, the continuity of the service drives the revenue generation and is as important as preserving the data. Thus, a complete shutdown of systems is only a last resort.

The definition of the states for the FSM is outlined in Figure 5, which provides two examples on how policies are aligned with the operational context of a business and its priorities, even when the same attack occurs, i.e., as applied to ransomware events. The above examples illustrate policies for the two presented use cases, where both the e-health and e-commerce scenarios feature frequent backups, remediation actions, threat sharing, and notification of the end-user. In the case of e-commerce, remediation differs to reflect the operational context. Namely, the cloud hosting instance is isolated and services are mirrored with the last backup. All devices within the business’s network are also actively monitored to evaluate the ransomware spread and prevent further damage.

The showcased FSM models are automatically transformed into UML representations after they are saved. The generated UML model can be directly used to initialise the IR and is executed on a specific trigger in the PALANTIR platform.

### 5.3. Constraint Satisfaction Problem for Enabler Selection

In this section, we expose and justify the modelling of the constraint satisfaction problem by blueprinting the design of the SM component.

We define B as the Boolean set and M as the set of the *n* available deployment configurations for a security mechanism. In practice, a deployment configuration refers to the option to deploy a mechanism implementing an SC to a specific infrastructure. We can state that

#(M)=n

This set can be enumerated, as follows:

$$M={\left(m_{i}\right)}_{0\leq i<n}$$

To generalise this problem to multiple security mechanisms, we consider

$$M^{\prime }={\left(m_{i}\right)}_{0\leq i<n^{\prime }}$$

with $\#\left(M^{\prime }\right)=n^{\prime }$ and $M\subseteq M^{\prime }$, where *M’* represents all the deployment configurations of all security mechanisms.

To define a valid constraint satisfaction problem, we must define three main parameters: (i) a set of variables to be affected as a solution of the CSP; (ii) a set of interval domains for the variables requiring affectation; and (iii) a set of constraints that the affected variable should satisfy to evaluate a solution as acceptable. As the core of the problem is to determine which deployment configuration should be enabled, we define the set of variables to be affected as

(1) $$X={\left(x_{i}\right)}_{0\leq i<n^{\prime }}$$

where $x_{i}$ denotes whether the deployment configuration $m_{i}$ is to be enabled or kept disabled. We can therefore infer the definition domain of those variables: they can either be affected by a positive value (1) to express a requested deployment, or by (0) to express its rejection. We formalise this assertion as follows:

(2) $$D={\left(D_{i}\right)}_{0\leq i<n^{\prime }}=B^{n^{\prime }}$$

We now have to express the different requirements of the SM component as a set of constraints to be satisfied by each affectation of *X*, and construct, accordingly, a cost function to order all the valid affectations. The following sections outline how infrastructure-issued and SCs-issued constraints and the cost of capability are evaluated.

#### 5.3.1. Infrastructure-Issued Constraints

We first tackle the constraints related to the consumption of computing resources on infrastructure. We assume an infrastructure $I_{q}$ belongs to a set of infrastructures (denoted by I) and is modeled as a tuple of its computing characteristics and a set of Boolean values reflecting the supported deployment model. Currently, our infrastructure model accounts for (i) the number of CPU cores available, (ii) the amount of RAM available, and (iii) the persistent storage space available.

We also reflect on the different deployment models for specific mechanisms. In real-life scenarios, we aim to discriminate the infrastructures from cloud computing, edge computing, and on-premises location, as their different technical natures will encourage the developers of the mechanisms to produce different versions of their component, adapted to each specific type of infrastructure.

Reasoning in terms of resource limitation permits us to implement an approach featuring the backpack problem, known to be NP-hard and representing a suiting case for constraints programming usage.

One naive method to model infrastructure resource constraints consists in ensuring each component’s resource requirement does not exceed what infrastructure can provide. Therefore, we may think that for blocking the allocation of a mechanism to an unsupported infrastructure type, we should change its resource consumption to be unlimited, to ensure the constraint is systematically violated when the solver will consider such deployment. However, some infrastructures (such as public clouds) may be modeled with an unlimited number of resources as well, driving the solver to an unpredictable state when evaluating the constraints. We overcome this pitfall by adopting a two-fold approach: we first model infrastructure and components reflecting their resources on offer and demands, and then their compliance and compatibility with the deployment models.

We can now write

(3) $$I_{q}=<r_{q}^{cpu},r_{q}^{ram},r_{q}^{stg}>\in N^{3}$$

where $r_{q}^{cpu}$ stands for the number of CPUs provided by the infrastructure *q*, $r_{q}^{ram}$ for its level of RAM, and $r_{q}^{stg}$ for its storage capacity. Similarly, for all ${\left(m_{i}\right)}_{0\leq i<n}$, we adopt the following notation:

1. $r_{m_{i},q}^{cpu}$ indicates the amount of CPU needed on infrastructure if deployment configuration $m_{i}$ is enacted;
2. $r_{m_{i},q}^{ram}$ states the amount of RAM needed;
3. $r_{m_{i},q}^{stg}$ stands for the required storage capacity.

These values can be null if the deployment configuration $m_{i}$ does not operate infrastructure *q*. Therefore, if $m_{i}$ is mono-infrastructure, there is only one infrastructure $I_{q}$, for which $r_{m_{i},q}^{cpu}$, $r_{m_{i},q}^{ram}$, and $r_{m_{i},q}^{stg}$ are non-null.

Therefore, we can formalise the first set of constraints to assess which mechanisms $I_{q}$ can support for deployments. The first equations relate to resource consumption:

(4) $$\begin{array}{c} \sum_{0\leq i<n}x_{i}r_{m_{i},q}^{cpu}<r_{q}^{cpu}\\ \sum_{0\leq i<n}x_{i}r_{m_{i},q}^{ram}<r_{q}^{ram}\\ \sum_{0\leq i<n}x_{i}r_{m_{i},q}^{stg}<r_{q}^{stg} \end{array}$$

They can be read as the sum of resource requirements for one deployed mechanism that have to be less than what the infrastructure can provide.

We can generalise (Equation (4)) to all security mechanisms by considering not just the sole set of deployment configurations of a single mechanism, but all of them:

(5) $$\begin{array}{c} \sum_{0\leq i<n^{\prime }}x_{i}r_{m_{i},q}^{cpu}<r_{q}^{cpu}\\ \sum_{0\leq i<n^{\prime }}x_{i}r_{m_{i},q}^{ram}<r_{q}^{ram}\\ \sum_{0\leq i<n^{\prime }}x_{i}r_{m_{i},q}^{stg}<r_{q}^{stg} \end{array}$$

This gives us the first set of constraints for a given infrastructure. They have to be evaluated for every single infrastructure $I_{q}$ considered for deployment.

To ensure the uniqueness of the deployment of a security mechanism and prevent multiple deployment configurations from being enacted multiple times on different infrastructures, we pose the following constraint:

(6) $$\sum_{0\leq i<n}x_{i}\leq 1$$

$\sum_{0\leq i<n}x_{i}$ is valued 0 if the security mechanism has not been retained for deployment; it is set as 1 otherwise.

In line with H2020 PALANTIR requirements, we consider the three deployment models presented in Section 4.8: Cloud, Edge, and Lightweight. Each infrastructure supports one deployment model. As some mechanisms may be supported by a subset of deployment mechanisms, they may not feature deployment strategies for all available infrastructures, but only for the ones they are compatible with.

#### 5.3.2. Security-Capability-Issued Constraints

The detection and mitigation methods of a SC also impact the selection of the mechanisms to be deployed. In the following section, we refer to them as *security property*. To model how a configuration deployment contributes to a security property to be accepted, we introduce a set of coefficients, where p∈N denotes the number of security properties to be satisfied.

We can therefore introduce

$${\left({\left(c_{i,j}\right)}_{0\leq i<n^{\prime }}\right)}_{0\leq j<p}$$

with $c_{i,j}\in B$. Therefore, for each of the *p* security properties to be satisfied, we identify the following constraint:

(7) $$\begin{array}{c} \sum_{0\leq i<n^{\prime }}x_{i}c_{i,1}\geq 1\\ \vdots \\ \sum_{0\leq i<n^{\prime }}x_{i}c_{i,j}\geq 1\\ \vdots \\ \sum_{0\leq i<n^{\prime }}x_{i}c_{i,p}\geq 1 \end{array}$$

This equation signifies that, for each one of the *p* properties to be satisfied, there must be at least one deployment configuration contributing to it.

#### 5.3.3. Construction of a Cost Function

We require a cost function that reflects the cost of the deployment of security mechanisms.

Let *t* be the reference period as a parameter of the problem. If we denote $e_{i}^{\mathrm{license}}$ the license for the i-th deployment configuration, $e_{i}^{\mathrm{instantiation}}$ the instantiation cost, and $e_{i}^{\mathrm{hourlycost}}$ the hourly cost of exploitation, the reference cost for a deployment configuration is

(8) $$e_{i,t}=e_{i}^{\mathrm{license}}+e_{i}^{\mathrm{instantiation}}+te_{i}^{\mathrm{hourlycost}}$$

We, therefore, build the following cost function:

(9) $$C_{t}\left(X\right)=\sum_{0\leq i<n^{\prime }}x_{i}e_{i,t}$$

Our cost approach is to select the solution providing the minimum value of the cost function.

### 5.4. Billing Models for SecaaS Operation

To exploit security mechanisms as services, their usage should be measured to determine their cost of operation by the security service provider and how their developers should be attributed. For instance, the provider may be subjected to infrastructure and support provision cost while the developer should secure funding to meet licensing agreement with software dependency suppliers. To that extent, we have adopted billing modalities adopted by the cloud industry, and support three different types of models:

1. *On-subscription billing*: the first deployment of a mechanism results in the subscription of a general licence at a fixed cost; further deployments will not induce further payments.
2. *On-instantiation billing*: each time a mechanism is scheduled for deployment, the fixed cost is charged.
3. *Operation billing*: when a mechanism is deployed, a cost proportional to its operation duration is charged.

These three models can be combined to finely tailor the fit of the expenditures due by the provider and the developer. Since PALANTIR’s deployment models impose varying deployment environments, SCs may differentiate in their implementations and infrastructure resource requirements. Therefore, each mechanism endorses its own parameterisation of the billing model. In practice, the data model exploited by the PALANTIR’s SCC stores a reference to (i) the supported deployment model, (ii) a subscription, (iii) an instantiation cost, and (iv) a hourly cost for each referenced SC implementation.

As the SCs are designed and their operation supervised by third parties, the subscribers require protection in case the service they deliver is hampered. A service-level agreement specifies a tolerated service downtime and the refunding modalities in case this duration is exceeded. To that end, the catalogue also accounts for those parameters in the SCs’ data model.

### 5.5. Security Capability Entry Model

Aligned with the above content, PALANTIR defines a data model for SC entries in the catalogue.

Essentially, the SCs are represented with two descriptors: the xNF descriptor and NS descriptor. The declaration of these descriptors, defined in NFV-SOL006 standard [57], includes several fields necessary for PALANTIR to identify SCs, namely, the *id*, *provider*, *version*, *product-name*, and *product-info-description* fields. These fields are exposed by the SO to the rest of PALANTIR platform.

Furthermore, PALANTIR extends the NFV-SOL006 standard to model the SC entries in the catalogue with the following aspects:

1. *Qualification of relevant SC:* PALANTIR defines some fields to classify the type of SC to make clear decisions as to where a new SC must be deployed, derived from the ontology presented in Section 5.1. These are *detection-method*, *mitigation-method*, *control-data-type*, and *monitor-data-type*.
2. *Billing of SC usage:* To define the requirements presented in Section 5.4, PALANTIR proposes *billing_model*, *subscription_billing*, *instance_billing*, and *hourly_billing* fields. Each SC has associated a different cost and a billing model regarding its capacities and characteristics.
3. *Assessment of the service-level agreement:* In this case, PALANTIR categorises the SCs with their service-level agreement applied and the violation fee associated. For this, *sla* and *sla_violation_fee* fields are added.
4. *Support for multiple deployment models:* The SC entry includes some fields to indicate the supported deployment model(s), and the resources needed to the correct SC operation. Therefore, the fields *deployment_model*, *nbCpu*, *amountRam*, and *amountStg* are defined.

Finally, PALANTIR takes the importance of GDPR compliance and proposes some fields to be in accordance with such law: (i) *interfaces_descr* to explain the nature of input/output traffic and the type of data used by the SC; (ii) *gdpr_applicability_descr* to describe the personal data manipulated, if applies; (iii) *storage_descr*, *processing_descr* and *sharing_descr* to inform about the storing, processing, and sharing of client data; (iv) *subject_right_descr* to allow stakeholders to indicate the rights associated (right to access, right to be forgotten, etc.) with the data used; (v) *open_internet_descr* to indicate whether SC impacts the respect on Open Internet norm; (vi) *non_discrimination_descr* to explain how data are used to discriminate users; and (vii) *eprivacy_descr* to detail the compliance with ePrivacy framework. We detail, in Table 1, the different fields composing the SC entry model.

## 6. Validation

We have previously presented the different PALANTIR building boxes enacting the remediation capacities of the platform, and delved into some key modelling aspects blueprinting them. In this section, we expose our evaluation plan to gauge their contribution to benefits of the SecaaS paradigm. We first present our findings from a qualitative evaluation, performed by analysing the implementation work conducted for the presented components; we then propose a quantitative evaluation to gauge the suitability of our approach in different conditions of operation.

### 6.1. Testbed Environment

In the two following subsections, we present two evaluation environments aligned with the two motivating cases presented beforehand.

The examples given for the remediation policies on a ransomware event (Section 4.2) are applied to the environments related to the use cases. It is worth noting that these take place once the threat is identified by other PALANTIR components (whose behaviour can be consulted in works such as [58,59]. At that point, the remediation procedure for a ransomware threat assumed in this use case leverages some other PALANTIR components, namely, from IR to SM, then to SO, and eventually to each SC. Specifically, the IR is notified about the threat and detects the best fitting actions. It then instructs the SM on which type of SC should be applied. The SM extracts information on the specific SC to deploy (i.e., Wazuh), as well as on the infrastructure’s constraints and billing options. Then, the SM instructs the SO to deploy the SC (achieved by retrieving its image from the local image registry and syncing from the SCC.

#### 6.1.1. Use Case 1: On-Premises Testbed for E-Health Environment

**Figure 6:**
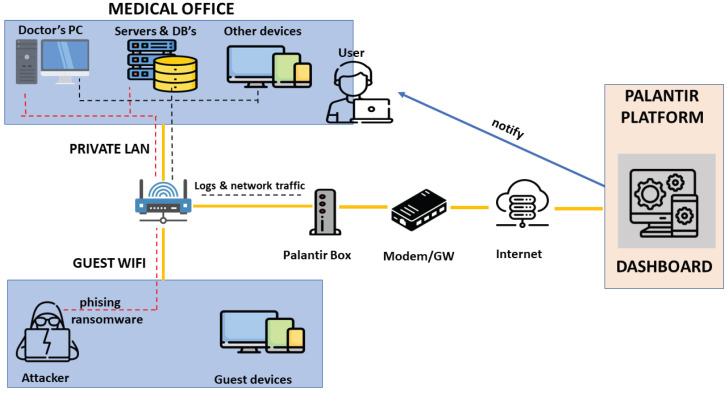
The testbed architecture of use case 1.

The testbed infrastructure for the protection of on-premises medical environments (e.g., medical offices) is presented in Figure 6. The Lightweight deployment model aligns with this case. Under the provisions of such model, a standalone CPE HW equipment is purposely prepared to support the baseline PALANTIR security and detection capabilities. The equipment is scaled according to the requirements imposed by the hosted PALANTIR components and limited by the requirements of the Lightweight deployment mode; which features a lightweight, reduced footprint device. However, depending on the actual size and scale of the medical office’s network, more advanced CPEs may be available. The CPE installed on-premises at the medical office is able to function in a standalone manner, without exposing local data. The only implemented interconnections are solely relevant (i) to manage and monitor the status of the CPE hosting the PALANTIR components; and (ii) to share detected attacks that may be related to other end-users.

The testbed topology at the medical office is split into two zones: (i) the office equipment and users and (ii) the guest network where the customers are connected. The CPE lies past beyond these two networks, connecting them with the modem/gateway for internet access. At the medical office, the critical assets are (i) the Medical Records database, withholding all patient-related data (e.g., personal data related to operations, medicines or exams); and (ii) the equipment used for specific exams, which is connected to the local network in order to provide access to the results (e.g., ultrasound or X-ray equipment). The above setup may be categorised as a cyber-physical system. Under the provisions of this use case, this environment is prone to attacks that aim at compromising the network and networked assets; in the case of ransomware attacks on the medical data stored in the medical database, the data leakage of sensitive patient information or the generic availability attacks can deteriorate the ability to use networked assets or access remote services.

The CPE (referred to as the "Palantir Box" in the middle of Figure 6) hosts the PALANTIR components that provide the baseline protection for the services in the medical office. The CPE inspects all incoming traffic and, in addition, logs from various monitored network nodes such as servers, PCs, and medical devices. When a particular type of attack is detected, specific measures are applied to mitigate it. In addition to this, the security service provider will be also notified of the incident and the end-user will also receive an additional notification.

#### 6.1.2. Use Case 2: Cloud Testbed for E-Commerce Platform

**Figure 7:**
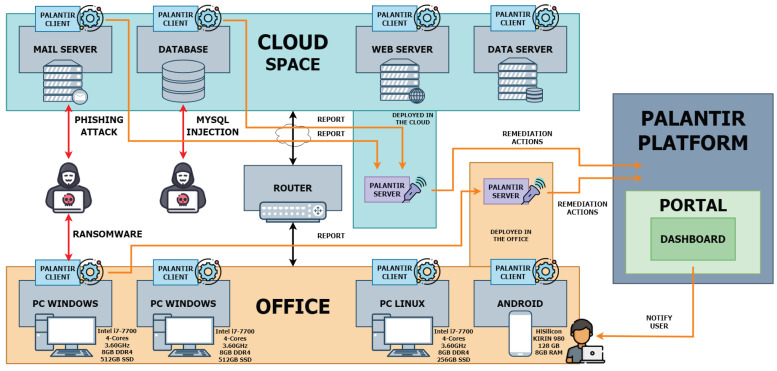
The testbed architecture of use case 2.

The testbed infrastructure for use case 2 is divided into three main parts. First, the Office, which represents the real SME office with Windows and Linux-based OS and Android smartphone. These devices are connected to the office router, which is also a gateway to the internet and the cloud space. Use case 2 deploys PALANTIR Wazuh Clients, which are services on the end devices, together with a PALANTIR Wazuh Server, which is another type of service that waits for messages from the PALANTIR Wazuh Clients and decides whether needs to forward them to the PALANTIR Platform. Each device is running the PALANTIR Wazuh Client as a service that tracks and collects the events and logs and shares those logs with the PALANTIR Wazuh Server, which can be set up locally on the premises or remotely. Figure 7 outlines that PALANTIR Wazuh Server is deployed locally on the premises to perform monitoring activity. The second part of the testbed represents the cloud space, where the SME hosts its mail, web, and data server together with the database. In the cloud space, PALANTIR Wazuh Clients are deployed and assigned the same task as devices in the SME office. The third part is the cloud deployment of the PALANTIR Ecosystem, which provides the remediation actions for detected cyber threats.

This use case features two PALANTIR deployment models: the Cloud deployment model to cover the “Cloud Space” part and the Lightweight deployment model to cover the “Office” part.

### 6.2. Qualitative Evaluation

We cover our implementation work for the proof-of-concept prototype of the components compounding our PALANTIR reference architecture for remediation. In the following subsection, we expose the technical specification of each component and justify how they contribute to the high-end objectives of our work.

#### 6.2.1. Incident Response Prototype Implementation

An incident response engine is a PALANTIR-protected infrastructure that hosts IR instances that are responsible for triggering mitigation policies when a threat/attack related to a data breach is detected. The objective of the IR is to handle remediation policies for threat mitigation, especially those that cannot be handled by the, for instance, cross-system or stakeholder notifications, which may be specific for an individual entity.

The IR component is implemented using Java language (version 11 of the OpenJDK) as a Spring Boot application. The Spring Boot application includes and runs the Apache Camel 3.9.0, Swagger (webjars) 3.51.2 and Spring Statemachine 2.0.0 dependencies. Apache Camel provides easy routing between synchronous (HTTP-REST) and asynchronous (Kafka) protocols. It also allows integration with other Java libraries. Swagger dependency prepared for Camel REST Domain Specific Language (DSL) provides simple setup and configuring of the REST endpoints for REST API. Once the Swagger is built and configured, it provides a Swagger UI interactive documentation accessible over the embedded Tomcat HTTP web server. Spring Statemachine is a framework for application developers to use state machine concepts with the Spring Boot application. Spring Statemachine framework runs the predefined Finite State Machine from the UML file. FSMs are defined graphically inside the Eclipse Papyrus modelling environment.

API platform that allows to creation and use of APIs. Postman streamlines collaboration and simplifies each phase of the API life cycle, allowing to design of better APIs quicker. We combine the use of Postman and Swagger UI when building and testing REST endpoints. The Swagger Specification is a REST API description format. API specifications are available in YAML and JSON formats.

The environment used for modelling and the creation of the FSMs is Eclipse Papyrus. Eclipse Papyrus provides a simple “drag and drop” workflow that exports the final FSM model to the UML document containing the FSM configuration. This document is then used to build the Spring State Machine inside the Spring Boot Java application.

#### 6.2.2. Service Matching Prototype Implementation

We implemented a proof of concept prototype using Java language (version 11 of the OpenJDK) and in conjunction with the SPRING framework version 2.5.6. It integrates with the Kafka broker though the Spring-Kafka plugin of version 2.7.8. The registration of deployment solutions is leveraged via the JDBC connector for rational database management system while the Jackson data bind layer enforces the data-access object paradigm. The prototype runs as a daemon on the testbed environments. The selection process of the SM relies on constraint programming. Choco-solver framework 4.10.7 [60] served as the baseline for implementing the constraint satisfaction problem. Our implementation prototype utilises the default search strategy.

To optimise connectivity, the prototype caches the information gathered from the SCC and the SO and request updates on a periodic basis. Therefore, during selection process, the SM configures the model constraint satisfaction problem via stored information only relating to the tenant under consideration.

In its implementation, the SM is open and not bound to any type of detection and mitigation methods, by re-exploiting those provided to the catalogue. This facilitates the adoption of new methods by PALANTIR’s platform, contributing to its openness. Moreover, the support of multiple deployment models in the solving process enables the system to match the adequate level of pervasiveness needed to leverage the protection.

Finally, the selection of the most cost-optimised deployment solution also facilitates the cost control of the operated security enablers.

#### 6.2.3. Security Capability Catalogue Prototype Implementation

The SCC prototype is based on Quarkus 2.15 and is written in Java 11. The SC Registration, SC Search, and SC Metadata Access compose the functionality of the SCC and are exposed as a REST API. The SC Data Validation, the SC Package Maker, and SC Onboarding on SO are background tasks executed as threads within the SCC Service. The core of SCC is represented by the data model in use by the SCC’s MongoDB. It is equivalent to the data model in use by the REST API. The clients of this REST API are filtered based on the identified role. The role is identified based on the JSON Web Token (JWT), which is used for authentication of the client that interacts with the SCC. The SCC interacts with the user management of the Portal in order to achieve the appropriate user filtering.

The API allows for the registration of an SC, viewing a list of SCs, searching for SCs based on some parameters, updating of an existing SC, and deletion. The API layer is only concerned with REST resources and the basic data validation on the controller of each resource. Although it appears as though it is a simple set of CRUD operations, various intricacies have to be tackled separately. As such, there are modules formed as internal services in the context of a service facade, each dedicated to a specific set of operations.

Access to the MongoDB database is implemented via a persistence layer added as a Quarkus plugin, which integrates with the MongoDB client and allows for the management of records. In the case of the SCC, the quarkus-mongodb-panache plugin is used, and a Panache Mongo repository is generated as an intermediate layer between the database and the core logic modules. For complex query/search and text-based search functionalities, a custom implementation is written based on Quarkus, which prepares the appropriate search or complex query to be fulfilled by MongoDB. The data inserted in MongoDB are always indexed, and some key SC metadata are indexed as a compound text index. The data indexing is configured in the SCC service via LiquiBase, instructing MongoDB to create indexes. Thus, whatever the search function, there is no performance penalty due to more exhaustive searches.

#### 6.2.4. Security Orchestrator Prototype Implementation

The SO provides an extra logic orchestrating layer that eases the management of the security mechanisms, via its deployment and configuration, as well as its monitoring and the overseeing of the whole workflow. To that end, it favours the principles of (i) compliance with standards’ specifications and (ii) the maintainability and extensibility.

To meet the former, the SO relies on its integration with OSM (an ETSI NFV-compliant MANO) to manage all security enactments. Therefore, specifications such as ETSI GS NFV-IFA 031 [61], regulating the interfaces (reference points) for the NFV MANO, are already considered by OSM and also indirectly complied with by the SO, specifically, in its southbound interface. The SO, in its central position to the security enforcement, partially aligns with the ETSI GS NFV-SEC 013 [21] specification. On one hand, it does so by acting as the NFV Security Manager (NSM) that cooperates with the MANO and adds extra logic to operate on virtualised services, such as Virtualised Security Functions (VSFs) and NFV-based Infrastructure Security Functions (ISFs). On the other hand, it also relates to the workflow, since it is designed to internally (i) introduce few of security decisions during the security planning stage (i.e., isolation of physical server); (ii) convey security policies and configuration changes to the NS instances; as well as (iii) introduce extra monitoring that can be used by other components in the PALANTIR architecture to plan further security actions. In addition to this, the SO also interacts with the VIM and xNFs using the expected reference points, laid out in the architecture.

As per the latter, the SO seeks to follow best practices not only from the NFV environment, but also from the cloud, partially disaggregating its implementation into multiple interconnected services, yet preserving some common elements to maintain a compromise between resource usage and data consistency (a shared database for all related services) against decoupling. Summing up, it combines practices from the microservices and Service-Oriented Architecture (SOA) approaches. The chosen hybrid design results in the better separation of concerns, which eases the extension and maintenance efforts to developers and operators, as they can more quickly identify the location of specific logic, be it to extend with a new development or to trace the execution of the software. Likewise, the implementation choices promote widely used languages (Python 3.8.14), and the intra-service communication using RESTful APIs (via Python’s Flask, version 2.0.2) and inter-service communication using both RESTful APIs (via Python’s FastAPI, version 0.73.0) and message brokers (through Python’s Kafka, version 2.0.2). The deployment techniques also consider both development (Python’s venv—or virtual environments in version 20.0.17) and production-ready environments (Docker-based deployments in the latest version with docker-compose in version 1.29.2), with both tools being widely used and documented by the community. Finally, the design of the SO considers multi-tenancy, whose implementation will allow adaptation to the different deployment models and multiple deployment locations for the SO.

#### 6.2.5. Security Capability Prototype Implementation

To create an SC, several steps are needed: (i) Define the Docker image (xNF) with the protection mechanism software already installed and correctly configured for the specific deployment; (ii) Implement the Juju charm (helped by a Python library) with the implementation of the available Day-0, Day-1 and Day-2 actions into the Docker image (deployed as *Podspec* charm together with the Juju operator) and generate a .charm file with *charmcraft* command; (iii) Define the xNF and NS descriptors, including the .charm file to be deployed with the SO; (iv) Instantiate the NS, which finally deploys three pods into K8s, namely, a Juju model container, the Juju operator (triggering actions into the service container), and a main container that uses the Docker image initially created. These steps are used for atomic SCs, i.e., SCs with one Docker image containing the protection mechanism software. For SCs with more than one Docker image or container, it is necessary to use Helm. Helm allows complex deployments in K8s, and can be used by the SO to deploy SCs. For instance, this approach is used by the SIEM SC, composed of four Docker images. In this context, the first step is to define the Helm chart with the required resources, and the second step is to implement a changejJuju charm proxy to interact with the deployment created. This approach is transparent to the final user since the triggering of Day-0, Day-1 and Day-2 actions is performed in the same manner, disregarding the SC containing one container or more (thus, using Helm).

We have implemented different SCs to detect and mitigate threats. Currently, four types of SCs have been deployed: IDS and DPI (Snort and Suricata [62]), FW (Iptables), DC (NetFlow and Zeek [62]), and SIEM (Wazuh [63]). All SCs implement the SEM element described in Section 4.6, which is finally implemented with two technologies: (i) Juju, in charge of triggering the different actions from the SO; (ii) Filebeat, a log collector that sends the logs generated in the SC to the Kafka broker in a specific Kafka topic. Finally, the current implementation provides a user-friendly approach that is transparent (abstracting low-level details) and adapted to the different deployment models.

#### 6.2.6. Security Portal Prototype Implementation

The prototype Portal is composed of a front-end and a back-end component. The front-end is simply the implementation of the web application, while the back-end is responsible for functionalities such as user management, storage of historical data, notification ingestion, and coordination of communication with the rest of the PALANTIR components.

The front-end is a React.js application, and is designed following the guidelines of Material UI. It is meant to be a fast and lightweight application that any user can access through their browser. The front-end is served via a node.js server. The serving of this application, i.e., the node server, is coupled with the portal back-end, which encloses the node.js server for the web application in its environment. The front-end connects to the PALANTIR platform by authenticating through the user authentication endpoint of the back-end REST API. Data access is achieved via calling the back-end REST API with Axios, and live notifications are sent to the front-end via a Web Socket connection using Socket.io. The REST API exposed by the back-end is segregated based on the different concerns, mainly viewing data points or datasets, as well as some basic management options.

The back-end is implemented as a small collection of services, organised around a service implemented in Java 11 using Quarkus 2.15 framework. Quarkus was selected as it is easily extensible, uses best-of-breed patterns, technologies, and standards, and is lightweight. The Quarkus flavor used is the reactive one, with reactive data types provided by Mutiny used wherever necessary, while in other cases methods are annotated as transactional. The data storage for the back-end is achieved using a PostgreSQL 15.1 database. Connectivity with the database is achieved with the standard Quarkus extensions, using JDBC and Hibernate ORM for PostgreSQL.

All notifications and events meant to be viewed by the user reach the portal via a Kafka message bus. In order to facilitate connection with the PALANTIR data and event streams in Kafka, the back-end uses the Kafka connector that is based on the Smallrye reactive messaging. The back-end connects to the different Kafka topics that the platform is supposed to use, and has consumer handlers that use the methods of the message classifier service. The messages are processed in order to be stored, or further processed before storage, and are also sent to the connected users via the open Web Socket connections. This real-time alerting is completed with the Quarkus Web Sockets extension, so that an alert socket is created when a new client connects to it.

KeyCloak is used for user Identity and Access Management (IAM) as an external service, and it is connected to the back-end with the Quarkus KeyCloak Admin Client, the usage of which is wrapped in custom service implementations. The key user data are kept in KeyCloak, while further data regarding client organisations and their users, such as the past incidents and applied remediations, are kept in the PostgreSQL database mentioned earlier.

### 6.3. Quantitative Evaluation

Extending the first qualitative evaluation, which is a first approach to the evaluation of the perceived functionality of the components in the PALANTIR platform, the following evaluation tends to its quantitative aspects, being provided with results from programmatic procedures, and analyses the outcomes.

#### 6.3.1. Performance Evaluation of the Security Capability Selection Process

As detailed in Section 4.3, the SM component determines the optimal deployment plan, considering financial restrictions, which satisfies the security needs to cope with any given detected malevolent activity. To that extent, this component accounts for the available SCs to consider and the configurations of the infrastructure (i.e., the entities defining the deployment location for a given SC). However, increasing the number of SCs also increases the complexity on the constraint model to be generated. This may have a dramatic impact on the performance, since the constraints engine solver (part of the SM) eventually relies on heuristic search to find an optimal solution. The objectives of the following experiments are to (i) quantify the impact of the model complexity on the component performance; and (ii) determine a point of inflexion in the model complexity, suggesting a partition in our satisfaction problem model. In practice, these experiments will help us to understand (i) in which conditions the SM should be scaled up to maintain acceptable performance for the end-user; and (ii) when the data collected by the component should be distributed into separated batches to be processed iteratively.

We understand the complexity of our model as the required number of variables and constraints given a specified number of both SCs and infrastructures, the latter modelled as infrastructure configurations. Such configurations comprise the characteristics of the infrastructures, e.g., the available amount of RAM memory, CPUs, storage capacity and supported deployment models. The number of variables to be solved is directly proportionate to the number of available SCs and infrastructure configurations. Therefore, only one of these parameters needs to be modified to quantify the different complexities of the model. In this case, we chose to modify the number of available infrastructure configurations, but a similar approach could have achieved by modifying the number of available SCs.

Table 2 lists the number of deployment configurations populated in the model, depending on the number of infrastructure configurations submitted to the SM component and assuming a fixed number of 40 eligible SCs.

Our evaluation work was conducted on a HW comprising 8-core Intel(R) Core(TM) i7-8550U at 1.8 GHz, with 16 GB of RAM, Linux operating system version 5.15.81 and Java Runtime environment version 11.0.17. We simulated the SO and SCC components by mocking their interfaces, that is, by exposing JSON files mimicking their responses through an HTTP server. A local Kafka broker (version 2.7.1) and a PostgreSQL database management (version 14.6) were locally deployed as dependencies of the prototype for the SM component. For this evaluation, we have focused solely on the solving procedure and components, and we have limited our measurements to the context of a threat. We have therefore extracted the measurements by exploiting the Java ThreadMXBean and MemoryMXBean interfaces to retrieve memory and CPU time metrics, as well as the timeCount interface from the Choco solver to retrieve the duration of the solver execution. Our measurement process consisted in iterating 50 requests on the same prototype instance for each evaluated model complexity. However, the evaluation of each model complexity has been conducted on different instances to ensure independence across measurements.

**Figure 8:**
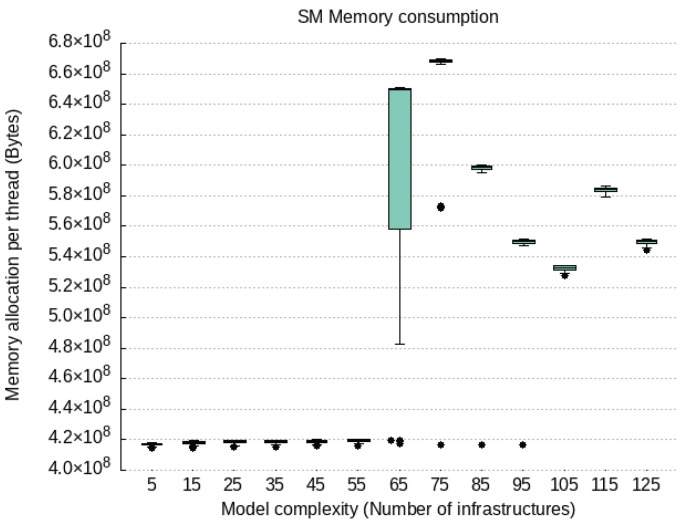
Measurements of thread memory consumption compared to constraints’ model complexity.

In our first experiment, we measured the heap RAM consumption depending on the complexity of the model, where Figure 8 exposes the results. We noticed a stable memory consumption profile for models with a complexity lower than 55 infrastructure configurations, but also that the memory consumption profile is not converging when the profile equals 65 infrastructure configurations. We also noticed that the profiles are converging (yet are not stable) for complexities exceeding 75 infrastructure configurations.

With this, we deduce that (i) there is an inflexion point when the complexity reaches 65 infrastructure configurations (or 2600 deployment configurations); and that (ii) increasing complexity of the model (surpassing this point of inflexion) does not translate into a systematic increase in memory consumption. We explain this behavior by the usage of the JVM’s memory saving mechanism, impacting the allocated structures of the search tree during the solving process.

**Figure 9:**
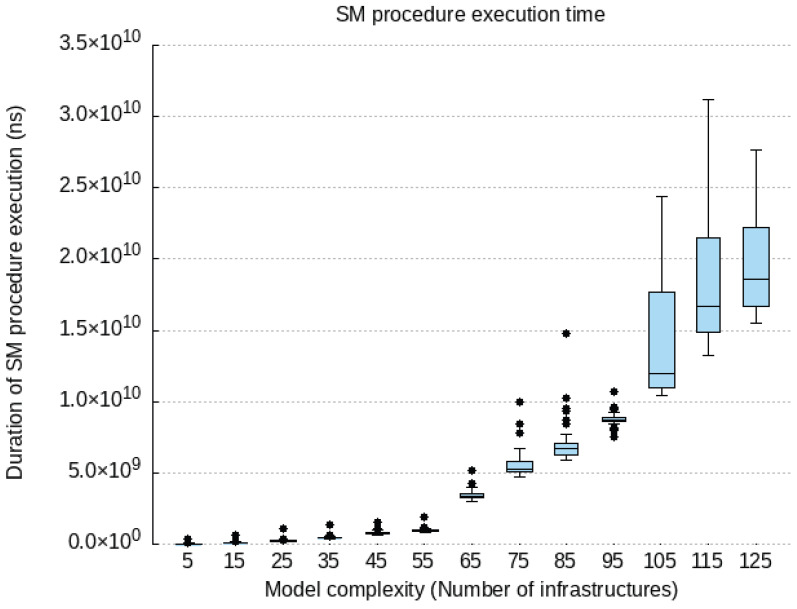
Measurements of SM procedure execution duration compared to constraint model complexity.

Our second experiment evaluates the duration of the execution of the main procedure of the SM component. This procedure is mono-threaded and comprises the interpretation of (i) the deployment requests, (ii) the population of the constraints model, (iii) its solving process, and (iv) the interpretation of the results. The distribution of the measured duration is illustrated in Figure 9. We confirm the presence of a point of inflexion once the model complexity reaches 65 infrastructure configurations, but also notice that the measurements are significantly less converging when the complexity exceeds 105 infrastructure configurations. We also notice the linearity of the duration distribution. Two tendencies can be isolated: during the first phase (when the complexity is limited to a maximum of 55 infrastructure configurations), adding one infrastructure’s configuration has only a marginal impact on the duration by only increasing the time by 18.2 ms; however, during the second phase (when the infrastructure complexity is between 75 and 125 infrastructure configurations), the impact is of 279.9 ms per added infrastructure configuration.

**Figure 10:**
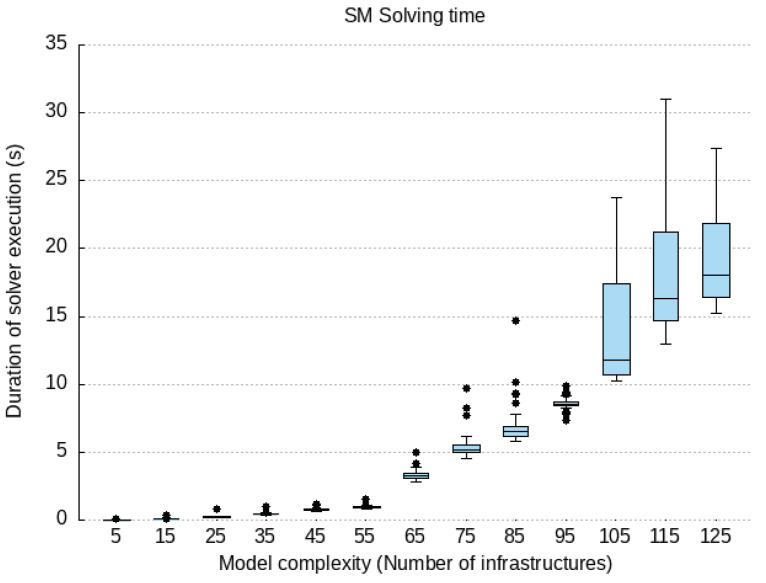
Measurements of solving duration compared to constraints model complexity.

In our last experiment, we investigated the specific duration of the solving process of the SM component, to understand the main reason for the increase in the duration. Figure 10 details our measurements. The obtained results conspicuously follow the same trends noticed in the second experiment, validating the same inflexion point and linearity of the distributions. We understand that the solving process is the main contributor to the duration of the process. In fact, adding an infrastructure in the first phase results in an average increase of 17.8 ms for the duration of the solving process; meanwhile, the second phase induces an addition of 276.6 ms per added infrastructure configuration.

Finally, Table 3 presents the mean and median values for each experiment.

#### 6.3.2. Orchestration of Security Capabilities

Given the central importance of the life cycle of the SCs by the SO, this was evaluated as an end-to-end process triggered from the SO, testing the three main life cycle operations which are heavily leveraged during the remediation process: (i) instantiation, (ii) reinstantiation, and (iii) configuration. Three of the four currently available SCs were tested, namely, iptnetflow, snort, and suricata.

Similarly, the measurements were extracted from a virtualisation environment managed by a K8s cluster, consisting of a control node along with two workers, both enforcing the default deployment setting (i.e., the workers allowed to schedule, the control one tainted). To evaluate the potential performance across different deployment scenarios, the K8s workers were first left running under full resources, and then constrained.

It is worth noting that this evaluation assumes a parallelism between the scenario running with full resources and the Edge and Cloud deployment models, given the increased availability of resources for such cases. In addition, the scenario with constrained resources relates to the Lightweight scenario, which features less specifications and is self-hosted.

The choice of the specific resources for the workers used in the constrained scenario is motivated by the current availability in the market of lower-end servers with such characteristics for a more modest price (in the range of 2k EUR), presumably more affordable for the microenterprises that would use it as the CPE for the Lightweight deployment model. Specifically, each of the two involved K8s workers were configured as indicated in Table 4, depending on the emulated environment.

In addition to this, the OSM instance (acting as the NFV MANO in the architecture) is set as a static VM in another server of the same testbed where the workers run, and it uses two CPUs (one socket with two cores), 8 GB RAM, and 60 GB disks to ensure no disruptions during the operation.

The evaluation process was conducted by running 100 times a Python script triggering all three life cycle actions from the SO and measuring its times in a sequential manner, so to ensure proper independence across tests. This process was repeated for each of the three analysed SCs. This all accounts for a total of 900 measurements for each of the 2 considered environments, plotted in the previous figures.

**Figure 11:**
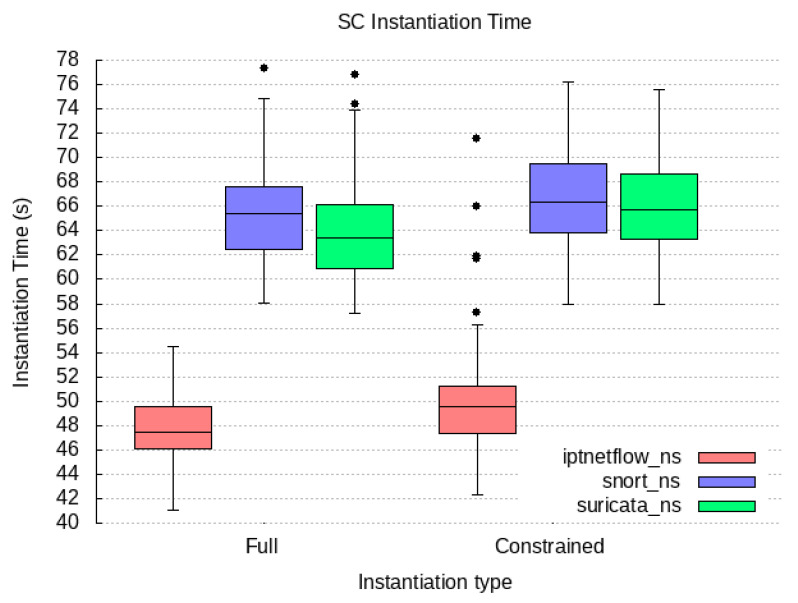
Distribution of instantiation times for the three devised SCs, comparing full (**left**) and constrained (**right**) deployments.

It can be appreciated that in Figure 11, the time taken to enact the operations in the full (left) K8s cluster varies with respect to the constrained (right) K8s cluster in that the median time taken by the latter accounts for up to 4.2% of the median time from the original instantiation tests.

**Figure 12:**
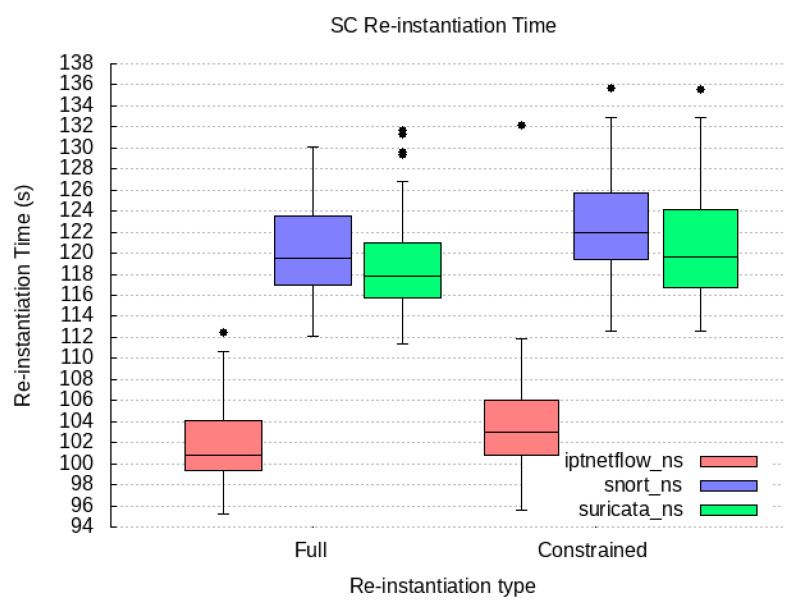
Distribution of reinstantiation times for the three devised SCs, comparing full (**left**) and constrained (**right**) deployments.

Likewise, Figure 12 shows similar differences when comparing the median times taken by reinstantiation, introducing a difference across such values up to 2.2%.

**Figure 13:**
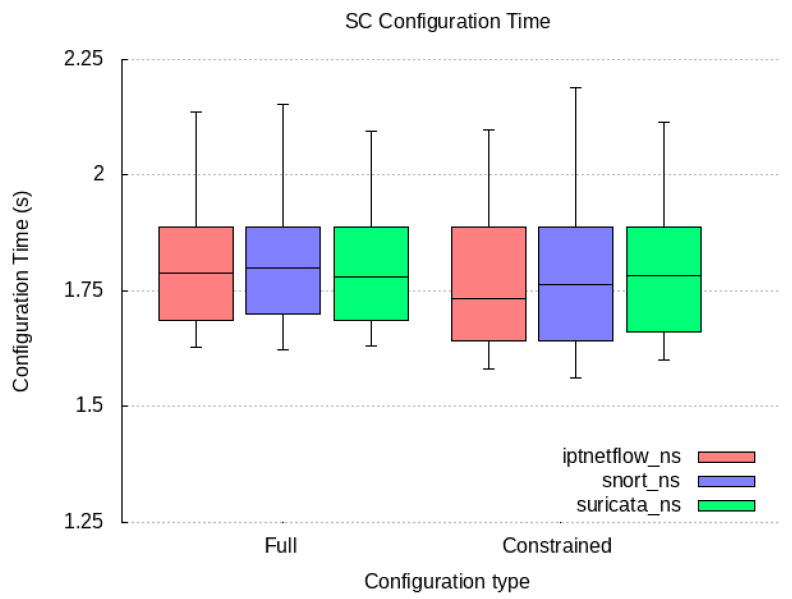
Distribution of configuration times for the three devised SCs, comparing full (**left**) and constrained (**right**) deployments.

In contrast to the clear pattern from the previous experiments, Figure 13 depicts a scenario with much less end-to-end time involved and less variability in absolute terms, but with the particularities of (i) the time distribution being more grouped in the scenario with full resources and; (ii) in average, counterintuitively taking the same or more time in the scenario with constrained resources.

The measurements above indicate that the instantiation and reinstantiation operations introduce slight time increase in the Lightweight scenario, where the scarcity of resources is more prominent, and similarly, where it is expected that less SCs will run concurrently.

A more straightforward comparison can be queried in Table 5, which extracts the mean and median values per experiment, environment, and type of SC. This produces a quick yet detailed comparison of the orchestration performance between an environment with the expected amount of resources, and a more limited one.

## 7. Conclusions and Future Work

In this paper, we present a security incident remediation strategy which targets cloud, edge, and on-premises environments to protect MEs and SMEs against an extendable set of cyber threats. A security ontology was presented and complemented by service-matching functionality delivered as a constraint-satisfaction problem. The functionality was used to determine the most pertinent security mechanisms by leveraging relevant cyber threats against the financial impact the deployment will impose on the business. We detail a policy format based on finite state machines to define and personalise an automated remediation procedure acting on the life cycle and local configuration of security mechanisms. We also propose three deployment models to ensure the adaptability and the pervasiveness of the deployment and operation of security mechanisms to different environments, as featured by two motivating cases in different verticals. We cover the blueprint of the security orchestrations supervising the life cycle of security enabler, highlighting the similarities with reference framework such as ETSI NFV MANO and SBI. We justify an extensive architecture enacting remediation procedures and implemented a proof-of-concept prototype utilised for quantitative evaluation of the performance of the remediation procedures in different deployment models.

Our work opens the door to several research opportunities. First, our ontology could be further refined to integrate specific properties of the resources to protect. We envision we could extend the service matching process to take this dimension into account, leveraging some new opportunities for remediation automation. In particular, accounting for the available monitoring information and configuration interfaces exposed by the resources needing protection could improve the selection of the technical security mechanisms to leverage. The implementation of security capabilities could be optimised to align with specific features of the resources, limiting their impact on the performance of the customer’s environment. Consequently, the composition of SC capabilities could also be regarded to cover a set of heterogeneous resources. Our ontology could be extended in that direction to introduce a dependency declaration, and the SM could evaluate their fulfillment. Moreover, the FSM-based model policy could be further extended to integrate a standardised interface with the security mechanism being configured. Integrating elements of the present ontology such as the methods of detection and mitigation could be the first step to drive this work. The main benefits of this effort would be twofold. From a security management perspective, this would contribute to decoupling the security logic from the local protection context, facilitating the response coordination against cyber threat in multi-site scenarios, at different levels of the cloud continuum. For the customers, avoiding the vendor lock-in would enable them to change their protection tooling with limited hassles to fit their evolving protection requirements. Finally, we believe our remediation framework could be complemented to enable not only reactive but also proactive threat management. Our ontology could be extended to cope with risk assessment and threat-sharing aspects, facilitating the exchange of information.

## Figures and Tables

**Table 1:** Security Capability entry model fields.

Name	Purpose	Description	Example
*id*	SO	ID to reference the SC	ids
*provider*	SO	Designer of the SC	Company
*version*	SO	Version of the SC	1.0
*product-name*	SO	Name of the SC	IDS SC
*product-info-description*	SO	Description of the SC	IDS for detection
*detection_method*	Extension	Detection method of the SC	network monitoring
*mitigation_method*	Extension	Mitigation method of the SC	network traffic filtering
*control_data_type*	Extension	Type of data generated by SC	NetFlow configuration
*monitor_data_type*	Extension	Type of data generated by SC	pcap
*billing_model*	Extension	Reference the billing models	hourly
*subscription_billing*	Extension	Price to subscribing	20.0
*instance_billing*	Extension	Price to deploy SC	1.0
*hourly_billing*	Extension	Fees per hour	0.1
*sla*	Extension	Amount of tolerated downtime per year	8 hours
*sla_violation_fee*	Extension	Discount by untolerated sla	0.1
*deployment_model*	Extension	Deployment model supported by SC	Cloud
*nbCpu*	Extension	Amount of CPUs used by SC	2
*amountRam*	Extension	Amount of RAM used by SC	2GB
*amountStg*	Extension	Amount of storage	20GB
*interfaces_descr*	GDPR	Explain the nature of data, input and output	Ingress and egress network traffic
*gdpr_applicability_descr*	GDPR	Explain data used	Anonymous IP addresses
*storage_descr*	GDPR	Explain storage purpose and techniques used	No storage
*processing_descr*	GDPR	Explain the data processing	Process blacklisted IP addresses
*sharing_descr*	GDPR	Explain the sharing of information with third parties	No third parties
*subject_right_descr*	GDPR	Allow users to establish rights to their data	Right to be forgotten established
*open_internet_descr*	GDPR	Respect the Open Internet norm	No traffic classification
*non_discrimination_descr*	GDPR	Explains how data discriminate users	Not applicable
*eprivacy_descr*	GDPR	Detail the compliance with ePrivacy	Not applicable

(Notation: SO, Extension, and GDPR, respectively, stand for SO requirements for SC operation, extension for SM and billing, and GDPR compliance.)

**Table 2:** Variation in the model complexity when comparing the number of submitted infrastructure configurations.

Number of Infrastructures Configurations	Number of Deployment Configurations
5	200
15	600
25	1000
35	1400
45	1800
55	2200
65	2600
75	3000
85	3400
95	3800
105	4200
115	4600
125	5000

**Table 3:** Summary of basic statistic measurements per experiment (in seconds).

	Thread Memory (MB)		Procedure Execution (s)		Solving Duration (s)	
**Complexity**	**Mean**	**Median**	**Mean**	**Median**	**Mean**	**Median**
5	416.9	417	0.0311	0.0235	0.019	0.016
15	418.1	418.4	0.1121	0.0941	0.097	0.086
25	418.6	418.9	0.2458	0.2186	0.228	0.209
35	418.7	419	0.4789	0.4387	0.453	0.424
45	419	419.1	0.7970	0.7711	0.759	0.742
55	419.5	419.7	0.9759	0.9499	0.933	0.919
65	600.7	649.4	3.4581	3.3699	3.325	3.25
75	653.8	668.4	5.5673	5.2830	5.408	5.142
85	594.9	598.6	7.0592	6.7032	6.903	6.525
95	547.4	550.2	8.8017	8.7383	8.603	8.539
105	532.5	533	14.0794	11.9839	13.815	11.768
115	584	584.5	18.5981	16.6595	18.316	16.289
125	549.5	549.9	19.5654	18.5535	19.238	18.066

**Table 4:** Virtual resources used per emulated deployment scenario.

	Sockets	Cores	RAM (GB)
Workers with full resources	3	4	20
Workers with constrained resources	1	4	8

**Table 5:** Summary of detailed statistic values per experiment, environment, and SC.

Experiment	Environment	SC	Mean (s)	Median (s)
Instantiation	Full	iptnetflow_ns	47.798	47.403
		snort_ns	65.609	65.419
		suricata_ns	63.811	63.361
		(Aggregated)	59.073	63.361
	Constrained	iptnetflow_ns	50.125	49.517
		snort_ns	66.748	66.342
		suricata_ns	66.587	65.695
		(Aggregated)	61.153	65.695
Reinstantiation	Full	iptnetflow_ns	101.576	100.747
		snort_ns	120.043	119.478
		suricata_ns	118.564	117.843
		(Aggregated)	113.394	117.843
	Constrained	iptnetflow_ns	104.049	103.054
		snort_ns	123.275	121.932
		suricata_ns	121.451	119.651
		(Aggregated)	116.258	119.651
Configuration	Full	iptnetflow_ns	1.816	1.788
		snort_ns	1.815	1.8
		suricata_ns	1.802	1.781
		(Aggregated)	1.811	1.788
	Constrained	iptnetflow_ns	1.793	1.739
		snort_ns	1.804	1.764
		suricata_ns	1.787	1.783
		(Aggregated)	1.795	1.764

## Data Availability

The several datasets generated for the qualitative evaluation procedures are openly available at https://github.com/palantir-h2020/paper-nfv-aas-threat-mitigation (accessed on 1 January 2023).
